# In Vitro and In Silico Insights into the Antiproliferative Activity of Sesquiterpenes from *Dittrichia viscosa* (L.) Greuter

**DOI:** 10.3390/ijms27157064

**Published:** 2026-08-06

**Authors:** Maria Michela Salvatore, Marco Masi, Muhammad Suleman, Haritha Kalath, Mai M. Karousa, Maha M. Ayoub, Abdullah A. Shaito, Anna Andolfi

**Affiliations:** 1 Department of Veterinary Medicine and Animal Production, University of Naples Federico II, 80137 Naples, Italy; mariamichela.salvatore@unina.it; 2 Department of Chemical Science, University of Naples Federico II, 80126 Naples, Italy; marco.masi@unina.it; 3 Biomedical Research Center (BRC), QU Health, Qatar University, Doha P.O. Box 2713, Qatar; m.suleman@qu.edu.qa (M.S.); harithakalath@gmail.com (H.K.); mk2200561@student.qu.edu.qa (M.M.K.); ma2400339@student.qu.edu.qa (M.M.A.); 4 Department of Biomedical Sciences, College of Health Sciences, QU Health, Qatar University, Doha P.O. Box 2713, Qatar; 5 College of Medicine, Qatar University, QU Health, Qatar University, Doha P.O. Box 2713, Qatar

**Keywords:** *Dittrichia viscosa*, tomentosin, inuviscolide, sesquiterpenes, network pharmacology, covalent molecular docking, anticancer activity

## Abstract

Four sesquiterpenes, namely tomentosin, 11α,13-dihydrotomentosin, inuviscolide, and α-costic acid, were isolated from the aerial parts of the medicinal plant *Dittrichia viscosa* (L.) Greuter and evaluated for antiproliferative activity against HCT116 colorectal and A549 lung cancer cells. All compounds reduced cell viability in a concentration-dependent manner, with tomentosin and inuviscolide exhibiting the greatest activity among the tested compounds and IC_50_ values in the moderate micromolar range. Evaluation in non-malignant human neonatal dermal fibroblasts (HDFn) revealed compound- and cancer-type-dependent selectivity, with inuviscolide showing greater selectivity toward HCT116 cells and tomentosin showing moderate selectivity toward both cancer cell lines. In silico ADMET and DFT analyses characterized the predicted drug-likeness, pharmacokinetic, toxicity-related, and electronic properties of the compounds, particularly tomentosin and inuviscolide. Network pharmacology prioritized STAT3, ESR1, and PTGS2 as candidate hub targets associated with both cancer types, while enrichment analyses identified pathways related to tumor proliferation, inflammation, immune regulation, and cellular stress. Noncovalent molecular docking, 200 ns molecular dynamics simulations, and MM/GBSA calculations supported possible modeled interactions of tomentosin and inuviscolide with these proteins, with tomentosin displaying the most favorable predicted binding profile. Covalent docking further identified structurally feasible STAT3 CYS687-linked poses with the two sesquiterpenes, although covalent adduct formation and direct target engagement remain to be experimentally validated. Overall, the integrated findings prioritize tomentosin and inuviscolide as *D. viscosa*-derived natural-product scaffolds with moderate antiproliferative activity for further structural optimization, selectivity assessment, and experimental mechanistic validation.

## 1. Introduction

The evergreen perennial plant *Dittrichia viscosa* (L.) Greuter is native to Mediterranean countries and Western Asia [1]. It is frequently found in anthropically altered environments, including the roadsides and ruderal habitats. With a long history in traditional medicine, *D. viscosa* provides effective remedies for both topical and internal applications [2]. 

Recent reports have shown the potential health benefits of organic extracts obtained from *D. viscosa* (synonym *Inula viscosa*) including an antiproliferative activity against human cancer cell lines, while showing minimal effects towards normal cells [3,4,5].

Due to its significant therapeutic value, several studies have been conducted to identify phytochemicals responsible for the bioactivities attributed to the plant. Previous investigations revealed that *D. viscosa* is rich in metabolites belonging to different classes of natural products, such as flavonoids, phenolic compounds and terpenoids. However, there is still limited knowledge about the specific contribution of individual metabolites to the bioactivities observed for *D. viscosa* extracts. Despite the growing interest in the metabolic composition of *D. viscosa*, most studies have focused on crude extracts [6,7,8,9].

Among the metabolites reported in *D. viscosa*, sesquiterpenes are particularly promising candidates due to their structural reactivity and preliminary biological evidence [7,10,11]. Several studies have reported the potential anticancer effects of sesquiterpenes and shown that the sesquiterpene lactones tomentosin and inuviscolide exert antiproliferative activity against human cancer cell lines [12,13]. The biological activities of sesquiterpene lactones have been largely associated with the presence of reactive α-methylene-γ-lactone moieties, which may interact with nucleophilic cellular targets and modulate pathways involved in inflammation, oxidative stress, apoptosis, and tumor progression [14]. Nevertheless, information regarding the anticancer potential of many *D. viscosa* sesquiterpenes remains limited, and direct comparisons among structurally related compounds isolated from the same plant source are scarce [12,13,15,16,17]. Accordingly, the present study provides a standardized side-by-side evaluation of four structurally related sesquiterpenes isolated from the same plant source and tested under identical experimental conditions, allowing direct comparison of their relative antiproliferative activities and structure-related differences within a single experimental framework.

**Figure 1:**
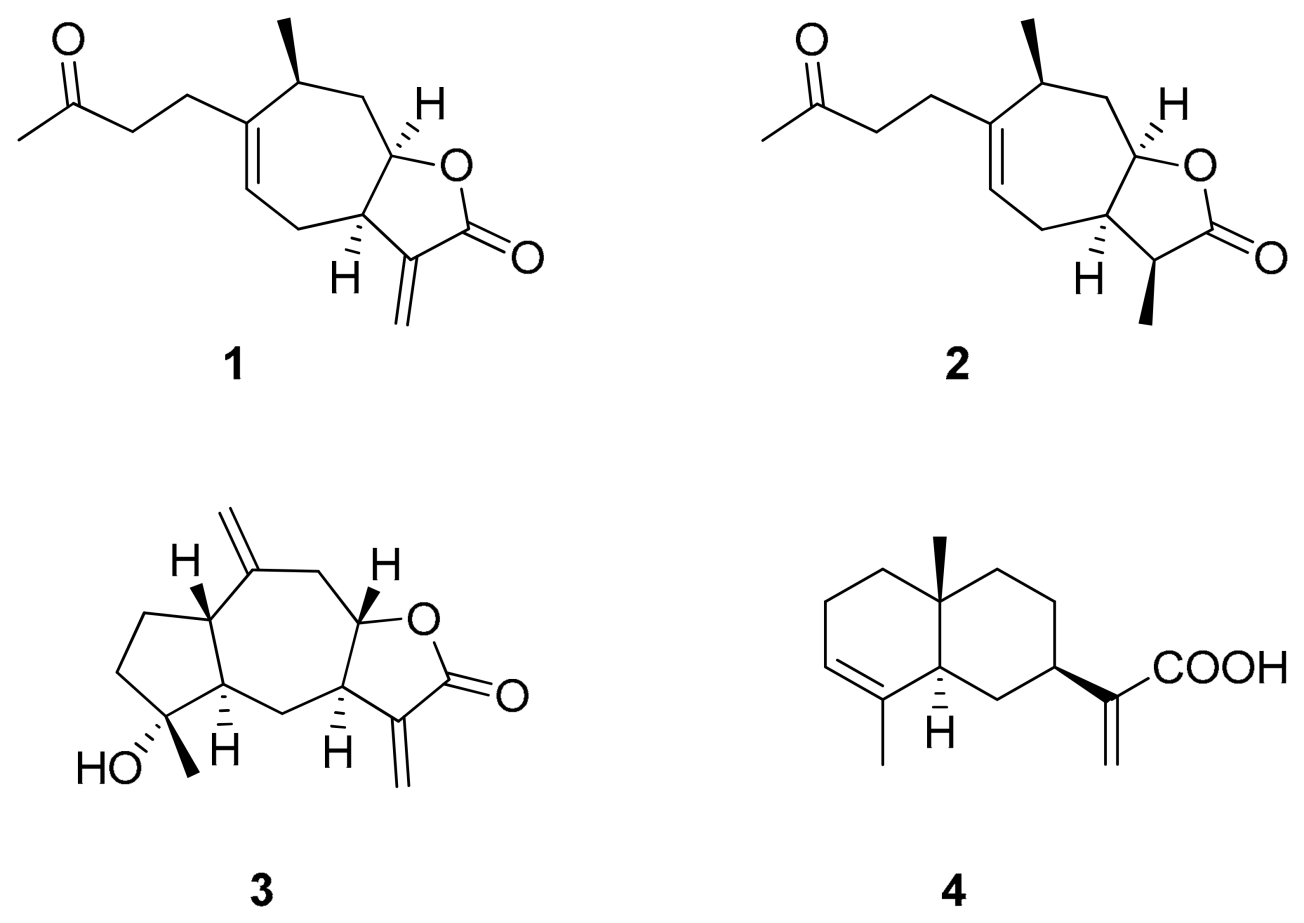
Structures of sesquiterpenes from *Dittrichia viscosa*: tomentosin (**1**), 11*α*,13-dihydrotomentosin (**2**), inuviscolide (**3**), and *α*-costic acid (**4**).

Therefore, this study aimed to comparatively evaluate the antiproliferative activity of tomentosin, 11*α*,13-dihydrotomentosin, inuviscolide, and *α*-costic acid isolated from *D. viscosa* (compounds **1**–**4** in Figure 1). Their effects were assessed in human colorectal HCT116 and lung A549 cancer cells, together with non-malignant human neonatal dermal fibroblasts (HDFn) to evaluate in vitro selectivity. Subsequently, an integrated computational workflow involving physicochemical and ADMET profiling, DFT calculations, target prediction, network pharmacology, protein–protein interaction network construction, functional enrichment analysis, molecular docking, 200 ns molecular dynamics simulations, and MM/GBSA calculations was applied to prioritize potential molecular targets and pathways associated with the observed antiproliferative effects. Based on the biological and network pharmacology findings, the most active compounds and prioritized hub targets were selected for further computational evaluation to characterize potential molecular interactions and guide future experimental validation. 

## 2. Results and Discussion

### 2.1. Sesquiterpenes from Dittrichia viscosa

Four sesquiterpenes have been isolated from the aerial parts of *D. viscosa* as reported in the Materials and Methods section and then identified via NMR, mass spectrometry and optical rotations by comparing the data with those reported in the literature [11,18,19,20] (see Figures S1–S18).

### 2.2. D. viscosa Sesquiterpenes Reduce the Viability of A549 and HCT 116 Cells

**Figure 2:**
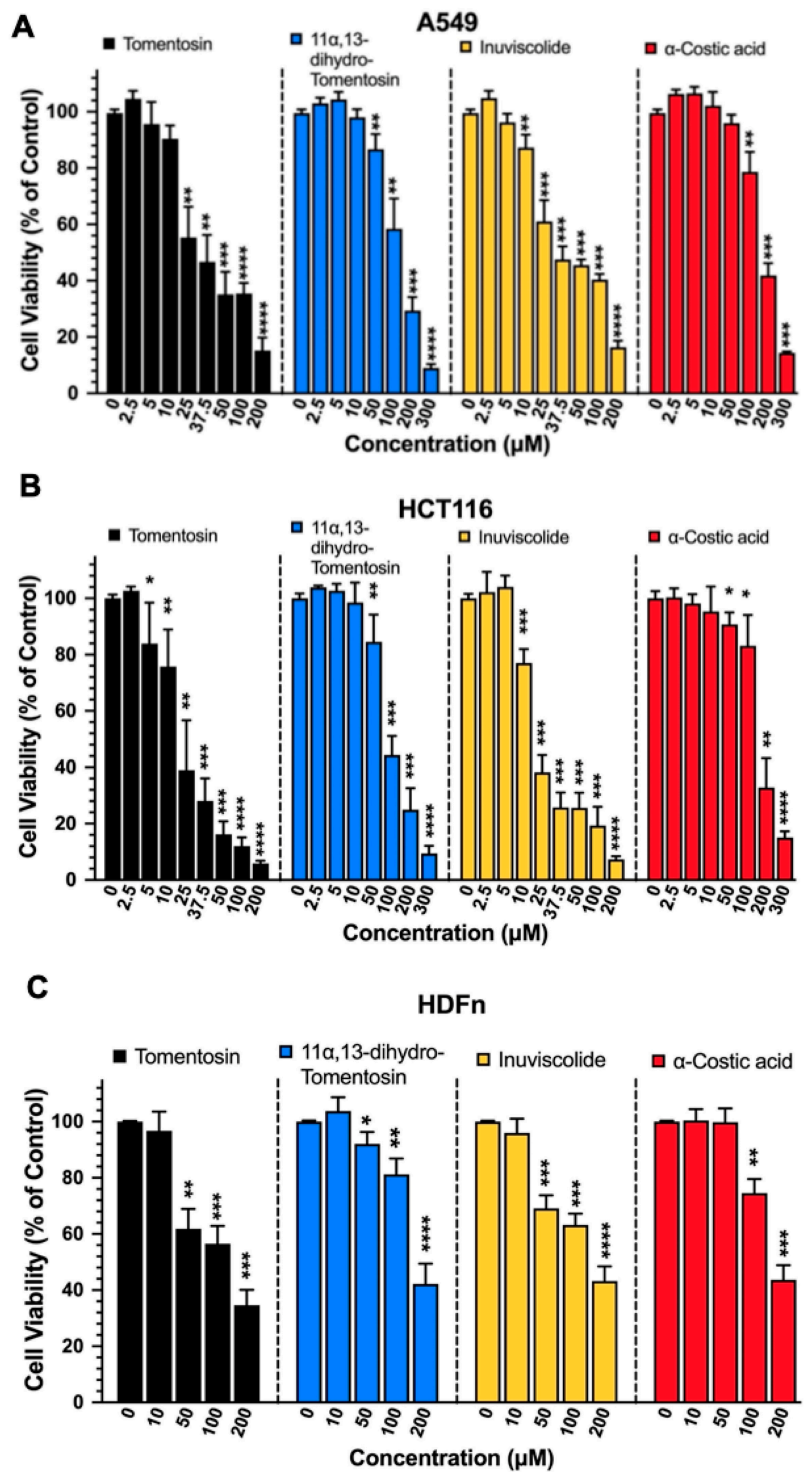
Effects of *D. viscosa* sesquiterpenes on the viability of HCT116 colorectal cancer, A549 lung cancer, and HDFn cells. A549 lung cancer cells (**A**) and HCT116 colorectal cancer cells (**B**) were treated for 48 h with increasing concentrations of tomentosin, 11α,13-dihydrotomentosin, inuviscolide, and *α*-costic acid (2.5–300 µM). Non-malignant human dermal fibroblasts, HDFn (**C**), were treated for 48 h with the same compounds at concentrations of 10–200 µM. Cell viability was assessed using the Alamar Blue assay and expressed as a percentage relative to vehicle-treated control cells. Data are presented as the mean ± SD of three independent experiments (*n* = 3). Statistical significance was determined relative to the corresponding vehicle-treated control: * *p* < 0.05, ** *p* < 0.01, *** *p* < 0.001, and **** *p* < 0.0001.

The antiproliferative activities of tomentosin, 11α,13-dihydrotomentosin, inuviscolide, and *α*-costic acid were evaluated against HCT116 colorectal carcinoma and A549 lung adenocarcinoma cells using the Alamar Blue cell viability assay. Following 48 h of treatment, all four sesquiterpenes reduced cell viability in a concentration-dependent manner, although to different extents (Figure 2). Tomentosin and inuviscolide exhibited the greatest antiproliferative activity among the tested compounds in both cell lines, whereas 11α,13-dihydrotomentosin and α-costic acid displayed comparatively weaker activity. The marked reduction in activity observed for 11α,13-dihydrotomentosin compared with tomentosin is consistent with established structure–activity relationships highlighting the contribution of the α-methylene-γ-lactone moiety to the cytotoxic and anticancer activities of sesquiterpene lactones [21]. 

HCT116 cells were generally more sensitive to treatment than A549 cells, as indicated by lower IC_50_ values for all compounds. In HCT116 cells, inuviscolide and tomentosin showed the best activity among the tested compounds, with IC_50_ values of 15.72 ± 3.81 and 17.82 ± 3.72 µM, respectively, while 11α,13-dihydrotomentosin and α-costic acid exhibited IC_50_ values of 86.84 ± 5.61 and 164.09 ± 7.62 µM. Similarly, in A549 cells, tomentosin and inuviscolide showed the greatest activity within the tested series, with IC_50_ values of 23.20 ± 1.26 and 28.63 ± 5.90 µM, respectively, compared with 149.27 ± 7.21 and 179.31 ± 8.90 µM for 11α,13-dihydrotomentosin and α-costic acid. Overall, tomentosin and inuviscolide showed the largest reductions in cancer-cell viability among the four sesquiterpenes and were therefore selected for subsequent molecular docking and molecular dynamics simulations to characterize possible interactions with computationally prioritized candidate targets. Potency classification depends on assay conditions including assay endpoint, exposure period, and experimental model. Historical NCI guidance used 4 µg/mL as an advancement benchmark for pure compounds, whereas NCI-60 uses 10 µM for initial screening rather than as a universal IC_50_ cutoff. Accordingly, tomentosin and inuviscolide are described here as showing moderate micromolar activity rather than high potency [22,23]. The activity observed for tomentosin is consistent with previous reports describing its cytotoxic, antiproliferative and pro-apoptotic effects in several cancer models [15]. 

The four sesquiterpenes were also evaluated in non-malignant HDFn cells after 48 h of exposure (Figure 2C). Tomentosin, 11α,13-dihydrotomentosin, inuviscolide, and *α*-costic acid exhibited IC_50_ values of 121.28 ± 8.36, 177.14 ± 9.58, 157.53 ± 12.36, and 176.54 ± 5.32 µM, respectively.

Tomentosin showed SI values of 5.23 toward A549 and 6.81 toward HCT116 cells, whereas inuviscolide showed SI values of 5.50 and 10.02, respectively. In contrast, 11α,13-dihydrotomentosin and *α*-costic acid showed SI values ranging from 0.98 to 2.04. These findings indicate compound- and cancer-type-dependent selectivity, with inuviscolide showing the highest selectivity toward HCT116 cells (Table 1).

The Alamar Blue assay provided a quantitative assessment of cellular metabolic viability and enabled a standardized comparison of the antiproliferative profiles of the four sesquiterpenes under identical experimental conditions. The present findings are therefore interpreted at the level of effects on cell viability and proliferation. Complementary apoptosis, cell-cycle, clonogenic, mitochondrial, and pathway-specific analyses will be valuable for further characterizing the cellular responses associated with these effects.

### 2.3. D. viscosa Sesquiterpenes Physicochemical, Drug-Likeness, and ADMET Properties

Evaluating the ADMET properties is essential in drug development, as it helps minimize safety risks, improve therapeutic efficacy, and support informed decision-making. Early evaluation of ADMET properties allows researchers to identify potential issues, optimize the compounds, and prioritize candidates with favorable pharmacokinetic and non-toxicity profiles [24,25]. Consequently, we predicted the physicochemical properties, lipophilicity, drug-likeness, and ADMET properties for the selected four compounds using the SwissADME and pkCSM pharmacokinetics online web tool as reported in the Materials and Methods section. The physicochemical properties such as molecular weight (MW), molecular refractivity (MR), number of H-bond acceptors (HBAs), number of H-bond donors (HBDs), and Topological polar surface area (TPSA) were evaluated for these compounds. All compounds satisfied the acceptable range of MW within 150–500 g/mol, HBA ≤  10, HBD ≤  5, and MR within the range of 40–130. Further, the compound lipophilicity, water solubility, Lipinski rule of five violations, and bioavailability were assessed to predict their potential as a drug candidate. All four compounds showed a Log P  ≤  5 value, with zero Lipinski rule of five violations, and a bioavailability score of more than or equal to 0.55. Among all the compounds, *α*-costic acid showed the highest bioavailability score of 0.85, suggesting potentially favorable drug-likeness and indicating its potentially higher rate of diffusion into the cell membrane and oral bioavailability, as depicted in Table 2. 

The permeability across the intestinal epithelium was assessed using the Caco-2 cell model, with all four compounds showing predicted human intestinal absorption values above 94%. The volume of distribution at steady state (VDss) parameter was computed to predict how extensively a drug distributes throughout the body and the total dose needed to achieve tissue concentrations like those in plasma. Compounds with log VDs < −0.15 L/kg are considered to have a low distribution, whereas those with VDs > 0.45 L/kg are categorized as having a high distribution. The VD predictions showed that among all the compounds, inuviscolide exhibited a higher distribution rate, 0.22 (log ml/min/kg), while others exhibited relatively lower distribution levels, with VD scores below 0.4 L/kg. Additionally, the compound interaction with cytochrome P450 (CYP) enzymes was analyzed to predict the metabolism of drugs in the body. The results indicated that all four compounds are non-inhibitors of the CYP2C19 enzyme. Notably, the excretion parameter total clearance values (log ml/min/kg) of all the compounds are higher than 1.0 log ml/min/kg, indicating their higher predicted elimination rates. Toxicity prediction includes parameters such as Ames toxicity and hepatotoxicity. The results suggested that none of the compounds studied showed Ames toxicity or hepatotoxicity, suggesting their non-mutagenic and safer profile for liver function, pending experimental toxicological validation. The ADMET properties for all compounds are presented in Table 2. 

### 2.4. Density Functional Theory (DFT) Analysis Reveals Favorable Electronic Properties of Tomentosin and Inuviscolide

**Figure 3:**
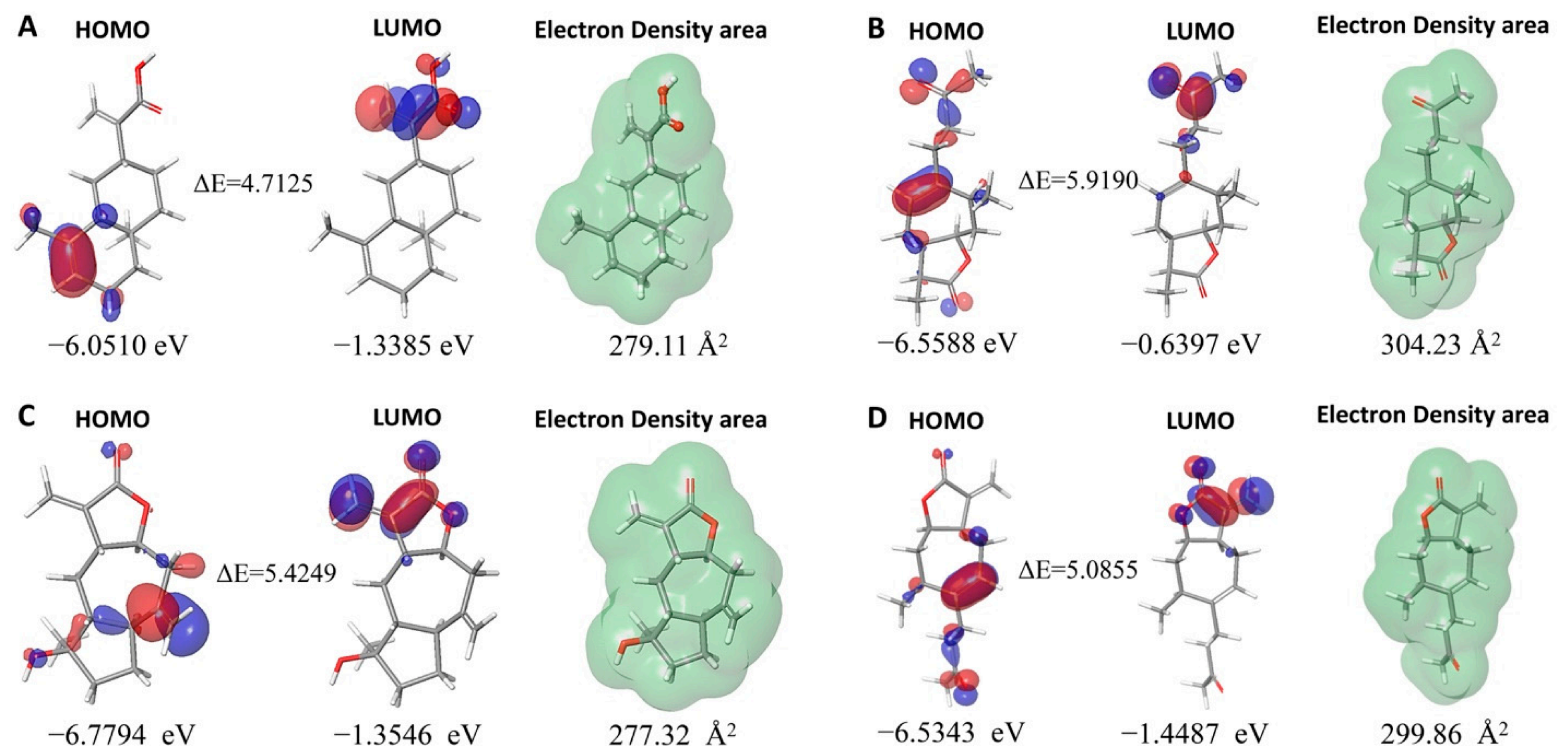
Density functional theory (DFT)-based frontier molecular orbital (FMO) analysis of the *D. viscosa* sesquiterpenes. Representative HOMO and LUMO electron density distributions and electron density areas (EDAs) are shown for (**A**) *α*-costic acid, (**B**) 11*α*,13-dihydrotomentosin, (**C**) inuviscolide, and (**D**) tomentosin. The FMO maps illustrate the electron-donating (HOMO) and electron-accepting (LUMO) regions of each molecule, whereas the HOMO–LUMO energy gap (ΔE) and EDA provide insights into their relative electronic reactivity and charge-transfer potential. The corresponding global quantum chemical descriptors derived from the DFT calculations are summarized in Supplementary Table S1.

Density functional theory (DFT) analysis provides valuable insights into the electronic structure and charge-transfer characteristics of small molecules, properties that may influence their interactions with biological targets. To further characterize the electronic properties of the *D. viscosa* sesquiterpenes, DFT calculations were performed for the four sesquiterpenes. The calculated global quantum chemical descriptors are summarized in Supplementary Table S1, while the frontier molecular orbital (FMO) maps are presented in Figure 3.

The four sesquiterpenes exhibited distinct electronic characteristics. *α*-Costic acid displayed the smallest HOMO–LUMO energy gap (ΔE = 4.71 eV), followed by tomentosin (5.09 eV), inuviscolide (5.42 eV), and 11α,13-dihydrotomentosin (5.92 eV), indicating relatively greater chemical reactivity for *α*-costic acid and tomentosin. This trend was further supported by their lower chemical hardness (η = 2.36 and 2.54 eV, respectively) and higher chemical softness (σ = 0.212 and 0.197 eV^−1^, respectively), suggesting an enhanced capacity for charge transfer during molecular interactions. In contrast, 11α,13-dihydrotomentosin exhibited the highest chemical hardness (η = 2.96 eV) and the lowest softness (σ = 0.169 eV^−1^), consistent with greater electronic stability.

Frontier molecular orbital analysis further distinguished the electronic behavior of the compounds. *α*-Costic acid possessed the highest HOMO energy (E_HOMO = −6.05 eV), indicating the strongest electron-donating tendency, whereas tomentosin exhibited the lowest LUMO energy (E_LUMO = −1.45 eV), suggesting enhanced electron-accepting capability. Inuviscolide displayed similar frontier orbital characteristics, while 11α,13-dihydrotomentosin showed the least favorable electron-accepting potential. The electrophilicity index (ω) was highest for tomentosin (3.13), followed by inuviscolide (3.05), *α*-costic acid (2.90), and 11*α*,13-dihydrotomentosin (2.19), indicating a greater propensity of tomentosin and inuviscolide to accept electron density during intermolecular interactions. Electron density area (EDA) analysis revealed that 11α,13-dihydrotomentosin possessed the largest electronic distribution, whereas inuviscolide exhibited the smallest. The FMO maps further demonstrated distinct HOMO and LUMO electron density distributions across the sesquiterpene lactone scaffolds (Figure 3 and Supplementary Table S1).

Overall, the DFT analysis demonstrated that tomentosin and inuviscolide possess favorable electronic characteristics, including relatively low HOMO–LUMO energy gaps (ΔE), moderate chemical hardness (η), high chemical softness (σ), and elevated electrophilicity indices (ω), which may facilitate charge-transfer processes during ligand–target interactions. In contrast, *α*-costic acid exhibited the greatest intrinsic chemical reactivity, whereas 11*α*,13-dihydrotomentosin was characterized by greater electronic stability. These quantum chemical characteristics are consistent with the observed in vitro antiproliferative activities of tomentosin and inuviscolide.

### 2.5. Prediction of Candidate Targets Associated with D. viscosa Sesquiterpenes and Lung and Colorectal Cancer

To obtain insights into the potential molecular mechanisms underlying the anticancer activities of the *D. viscosa* sesquiterpenes, a network pharmacology approach was employed to predict the molecular targets of tomentosin, 11*α*,13-dihydrotomentosin, inuviscolide, and *α*-costic acid. Bioinformatics-based target prediction provides valuable clues for identifying candidate therapeutic targets and exploring the multitarget pharmacological properties of natural products in complex diseases such as cancer. Compound-associated targets were retrieved from the SwissTargetPrediction and PASS Targets databases. SwissTargetPrediction identified 100 putative targets for each of the four compounds, whereas PASS Targets predicted 82, 89, 101, and 144 targets for tomentosin, 11*α*,13-dihydrotomentosin, inuviscolide, and *α*-costic acid, respectively (Supplementary Table S2). To identify candidate targets associated with the investigated cancer types, disease-associated genes were retrieved from Genecards, NCBI, and MalaCards databases, yielding 10,546 lung cancer-related genes and 10,674 colorectal cancer-related genes (Supplementary Tables S3 and S4, respectively). 

**Figure 4:**
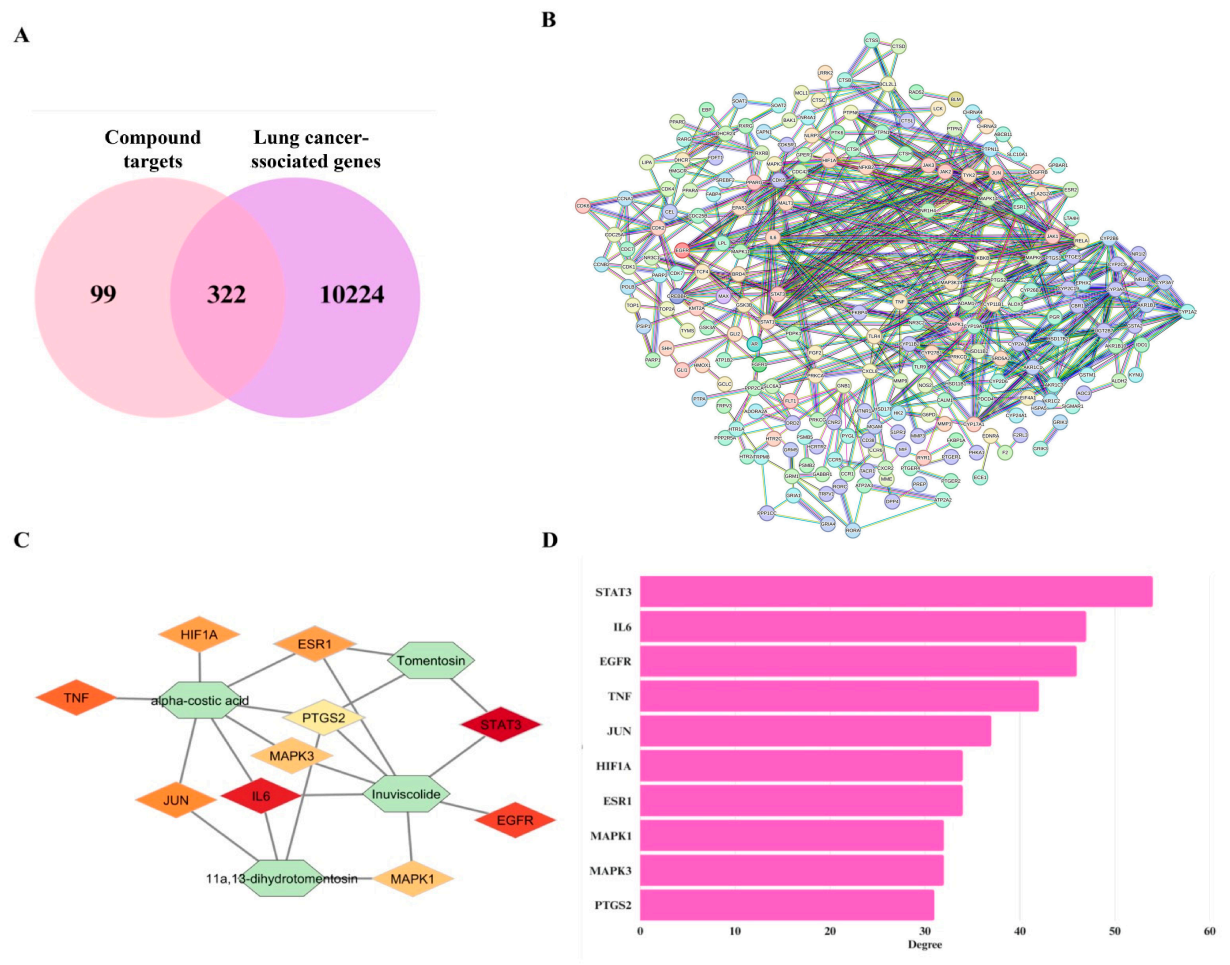
Network pharmacology analysis of *D. viscosa* sesquiterpenes and lung cancer-associated targets. (**A**) Venn diagram illustrating the overlap between predicted compound targets and lung cancer-associated genes. (**B**) Protein–protein interaction (PPI) network of the shared targets constructed using the STRING database. (**C**) Compound–hub gene interaction network showing the relationships between the four sesquiterpenes and the top 10 lung cancer hub genes identified by CytoHubba. The green nodes represent the investigated sesquiterpenes, whereas the hub genes are color-coded according to their degree centrality ranking, with red indicating the highest degree scores and yellow indicating the lowest degree scores among the selected hub genes. (**D**) Bar plot showing the degree scores of the top 10 hub genes associated with lung cancer.

**Figure 5:**
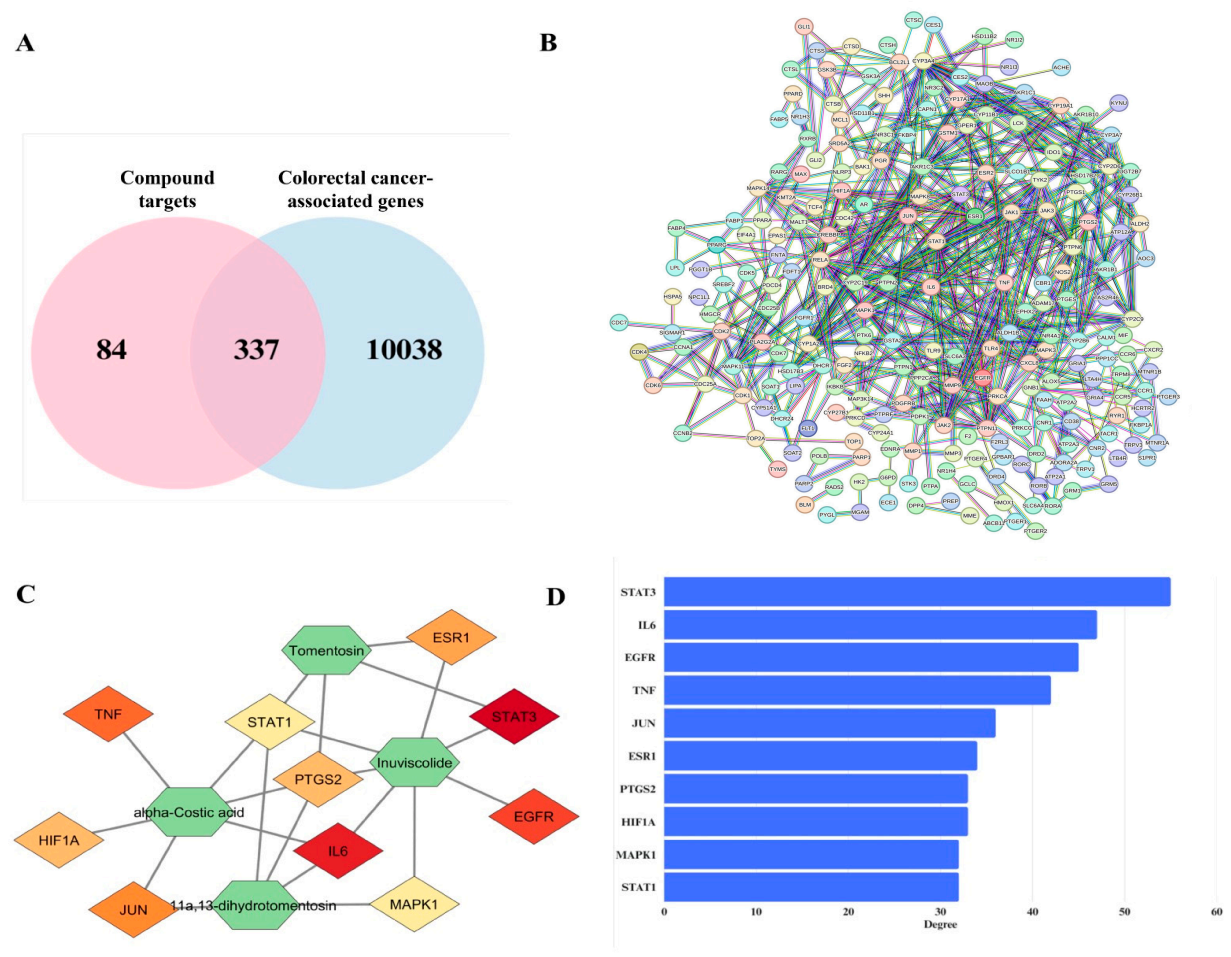
Network pharmacology analysis of *D. viscosa* sesquiterpenes and colorectal cancer-associated targets. (**A**) Venn diagram illustrating the overlap between predicted compound targets and colorectal cancer-associated genes. (**B**) Protein–protein interaction (PPI) network of the shared targets constructed using the STRING database. (**C**) Compound–hub gene interaction network depicting the interactions between the four sesquiterpenes and the top 10 colorectal cancer hub genes identified by CytoHubba. The green nodes represent the investigated sesquiterpenes, whereas the hub genes are color-coded according to their degree centrality ranking, with red indicating the highest degree scores and yellow indicating the lowest degree scores among the selected hub genes. (**D**) Bar plot showing the degree scores of the top 10 hub genes associated with colorectal cancer.

Overlapping of the compound- and disease-associated targets enabled the identification of common targets potentially involved in the anticancer activities of the *D. viscosa* sesquiterpenes (Figure 4A and Figure 5A). Venn diagram analysis revealed 322 overlapping targets between the predicted compound targets and lung cancer-associated genes (Figure 4A and Supplementary Table S5), and 337 common targets with colorectal cancer-associated genes (Figure 5A and Supplementary Table S6). These shared target genes represent computationally identified putative therapeutic mediators of the anti-colorectal cancer activities of the investigated compounds, pending experimental validation.

### 2.6. PPI Network Analysis Identifies Shared Hub Genes and Prioritizes Candidate Targets Associated with D. viscosa Sesquiterpenes

Protein–protein interaction (PPI) network analysis is a valuable network pharmacology approach for identifying key molecular targets and signaling pathways involved in disease pathogenesis and therapeutic intervention [26]. To characterize the functional relationships among the overlapping compound- and disease-associated targets, PPI networks for lung and colorectal cancers were constructed from the compound–disease overlapping targets (Figure 4A and Figure 5A) using the STRING database (Figure 4B and Figure 5B). The resulting networks were subsequently analyzed using the CytoHubba plugin to identify and rank the most highly connected hub genes based on degree centrality (Figure 4C,D). For lung cancer, the top 10 hub genes were STAT3, IL6, EGFR, TNF, JUN, HIF1A, ESR1, MAPK1, MAPK3, and PTGS2, with degree scores ranging from 54 to 31 (Figure 4 and Figure 5). Similarly, the colorectal cancer network identified STAT3 (55), IL6 (47), EGFR (45), TNF (42), JUN (36), ESR1 (34), PTGS2 (34), HIF1A (33), MAPK1 (32), and STAT1 (32) as the top-ranked hub genes (Figure 5C,D). Several hub proteins, including STAT3, IL6, EGFR, TNF, JUN, ESR1, PTGS2, HIF1A, and MAPK1, were shared between the two cancer types. Network pharmacology prioritized STAT3, ESR1, and PTGS2 as computational candidate hub targets shared between the two cancer-associated networks. Given their greater in vitro antiproliferative activity, tomentosin and inuviscolide were selected for downstream computational analyses. STAT3, ESR1, and PTGS2 were subsequently selected for molecular docking and molecular dynamics simulations because they were among the highest-ranked shared hub genes between the two cancer types and were predicted targets of both compounds (Figure 4C and Figure 5C).

### 2.7. Gene Ontology and KEGG Pathway Enrichment Analyses Identify Common Oncogenic Processes Targeted by D. viscosa Sesquiterpenes

**Figure 6:**
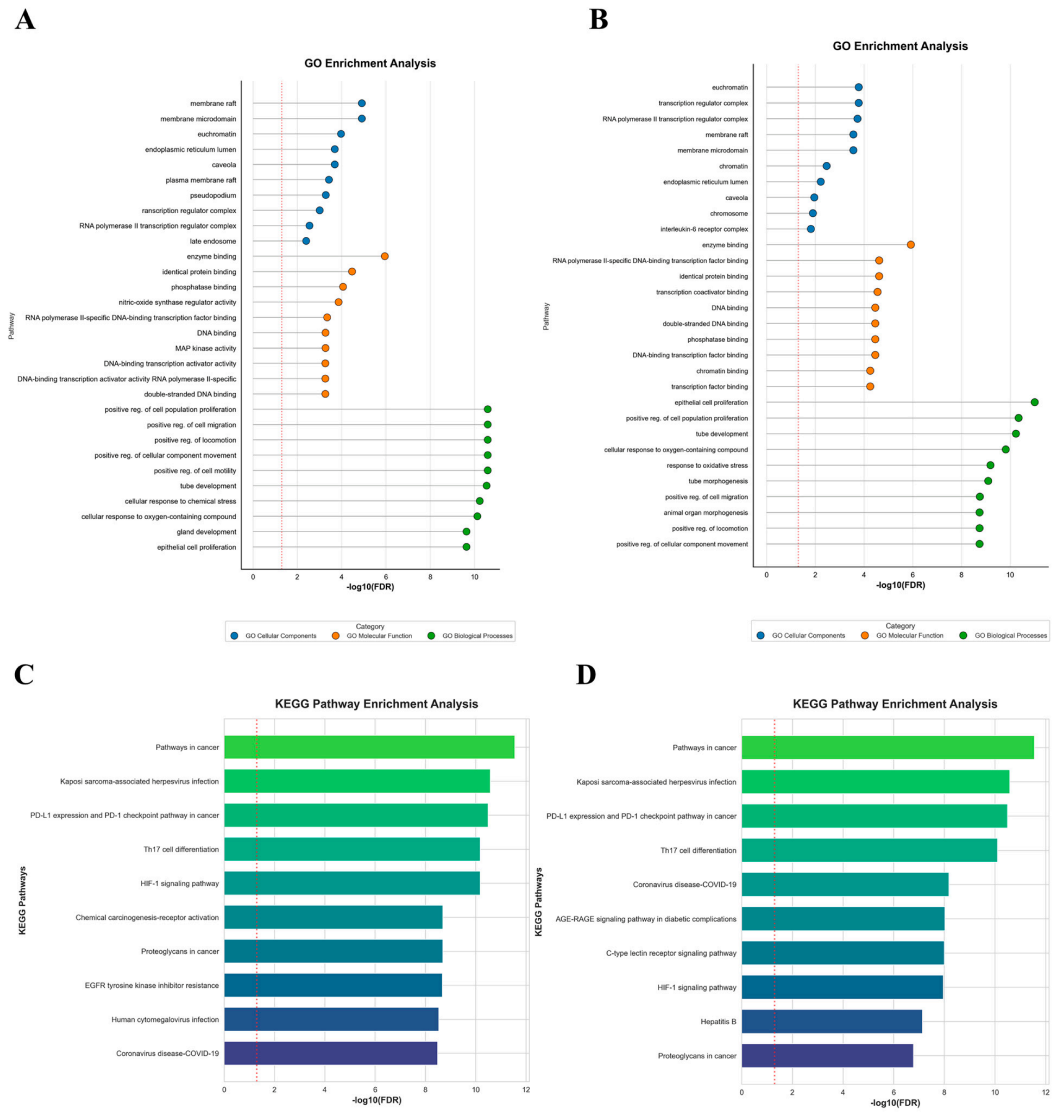
Gene Ontology (GO) and KEGG pathway enrichment analyses of the e compound–disease overlapping target sets associated with *D. viscosa* sesquiterpenes. Bubble plots depict the top significantly enriched GO terms ranked by −log10(FDR) (FDR < 0.05). (**A**) GO enrichment analysis of the full set of overlapping targets between predicted compound targets and lung cancer-associated genes. (**B**) GO enrichment analysis of the full set of overlapping targets between predicted compound targets and colorectal cancer-associated genes. (**C**) KEGG pathway enrichment analysis of the full lung cancer compound–disease overlapping target set. (**D**) KEGG pathway enrichment analysis of the full colorectal cancer compound–disease overlapping target set.

To gain further insight into the biological functions and signaling pathways associated with the identified hub genes, Gene Ontology (GO) and Kyoto Encyclopedia of Genes and Genomes (KEGG) pathway enrichment analyses were performed separately using the complete lung cancer and colorectal cancer compound–disease overlapping target sets through the ShinyGO web server. The top 10 significantly enriched GO terms were classified into Biological Processes (BPs), Molecular Functions (MFs), and Cellular Components (CCs), and are presented in Figure 6. For lung cancer, GO enrichment analysis revealed that the hub genes were predominantly associated with BPs involved in cancer progression, including the positive regulation of cell proliferation, cell migration, locomotion, and motility, as well as tube and gland development and cellular responses to chemical stress and oxygen-containing compounds. Enriched MFs included enzyme binding, identical protein binding, phosphatase binding, nitric oxide synthase regulator activity, RNA polymerase II-specific DNA-binding transcription factor binding, and DNA binding. CC analysis indicated significant enrichment in membrane rafts, membrane microdomains, euchromatin, and the endoplasmic reticulum lumen (Figure 6A and Supplementary Table S7).

A comparable enrichment profile was observed for colorectal cancer predicted hub genes (Figure 6B and Supplementary Table S8). The most significant BPs included epithelial cell proliferation, tube development, and cellular responses to oxidative stress and oxygen-containing compounds. Enriched CC terms comprised euchromatin, transcription regulator complexes, RNA polymerase II transcription regulator complexes, membrane rafts, and membrane microdomains, whereas the predominant MFs involved enzyme binding, RNA polymerase II-specific DNA-binding transcription factor binding, identical protein binding, transcription coactivator binding, and DNA binding. The substantial overlap in enriched GO terms between lung and colorectal cancers suggests that the computationally identified target sets are associated with common biological processes involved in tumor development and progression. 

To complement the GO analysis, KEGG pathway enrichment was performed to place the identified hub genes within broader signaling and disease-related networks. For lung cancer, the most significantly enriched pathways included “Pathways in cancer”, “Kaposi sarcoma-associated herpesvirus infection”, “PD-L1 expression and PD-1 checkpoint pathway in cancer”, “HIF-1 signaling pathway”, and “Th17 cell differentiation” (Figure 6C and Supplementary Table S7). Similarly, colorectal cancer hub genes were significantly enriched in “Pathways in cancer”, “Kaposi sarcoma-associated herpesvirus infection”, “PD-L1 expression and PD-1 checkpoint pathway in cancer”, “Th17 cell differentiation”, and “Coronavirus disease” pathways (Figure 6D and Supplementary Table S8).

Collectively, the GO and KEGG enrichment analyses indicated that the predicted hub genes potentially converge on key biological processes and signaling pathways involved in tumor proliferation, inflammation, immune regulation, and cellular stress responses. The shared functional and pathway enrichment profiles observed across lung and colorectal cancers further support the hypothesis that *D. viscosa* sesquiterpenes may exert their anticancer effects through a multitarget mechanism involving the coordinated modulation of interconnected oncogenic networks and may be employed to guide future experimental evaluation. 

### 2.8. Molecular Docking of Tomentosin and Inuviscolide Against Selected Hub Proteins

To further investigate the molecular basis of the observed antiproliferative activities, molecular docking studies were performed for tomentosin and inuviscolide, the two sesquiterpenes that exhibited the greatest cytotoxicity against HCT116 and A549 cells. Based on the network pharmacology analysis, STAT3, ESR1, and PTGS2 (COX-2) were selected as representative targets because they were among the highest-ranked hub genes and were identified as common predicted targets of both compounds. Reference inhibitors, including Stattic for STAT3, 4-hydroxytamoxifen for ESR1, and rofecoxib for PTGS2, were included to validate the docking protocol. The binding affinities and key protein–ligand interactions are summarized in Table 3.

### 2.9. Predicted Noncovalent Interactions of Tomentosin and Inuviscolide with the STAT3 SH2 Domain

**Figure 7:**
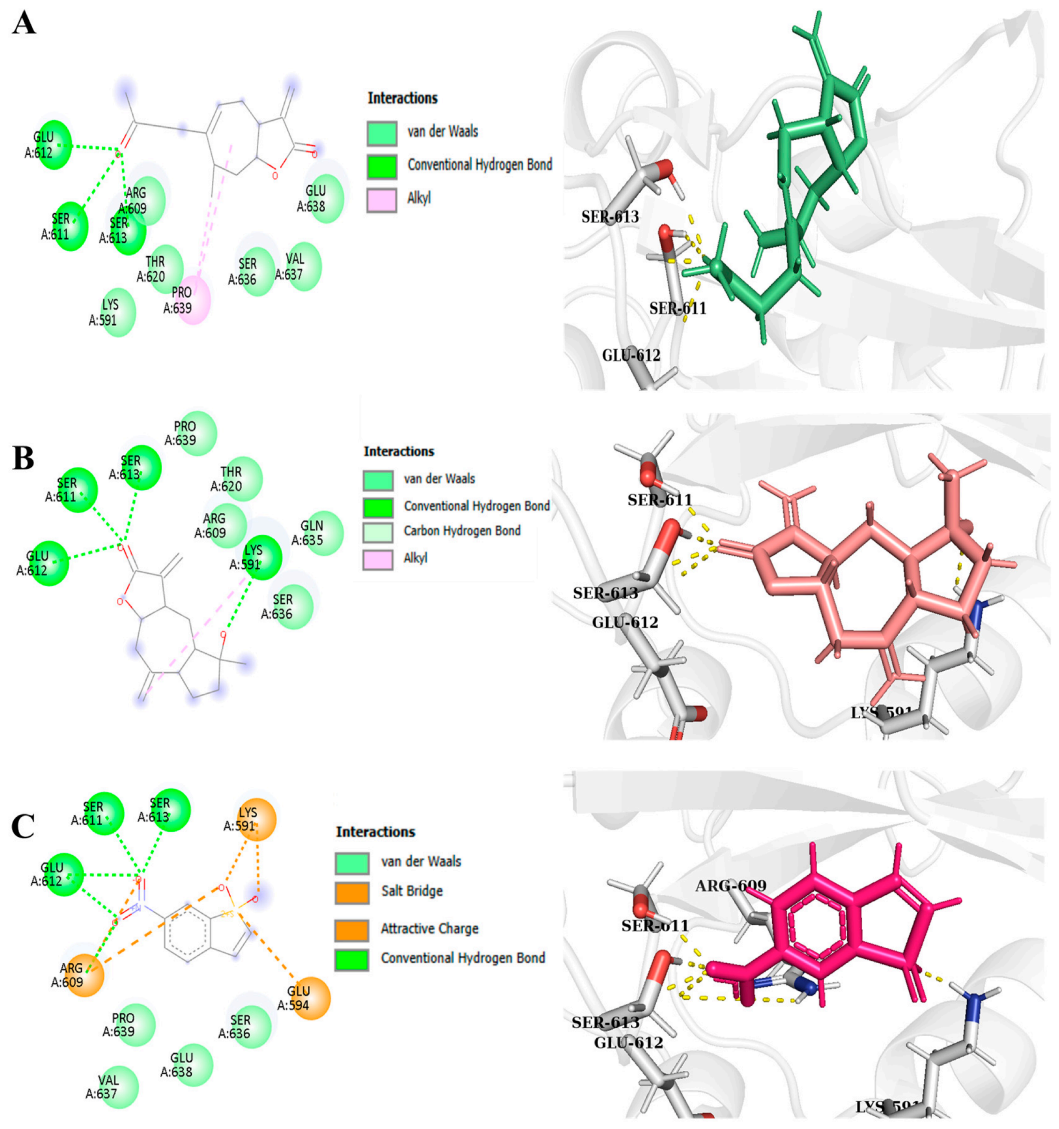
Molecular docking analysis of tomentosin and inuviscolide with the STAT3 SH2 do-main. Two-dimensional (left) and three-dimensional (right) representations of the protein–ligand interactions between STAT3 and (**A**) tomentosin (Green), (**B**) inuviscolide (Salmon pink), and (**C**) the reference inhibitor Stattic (Megenta). The interaction maps highlight key residues involved in ligand recognition, including hydrogen bonds, salt bridges, hydrophobic interactions, alkyl con-tacts, and van der Waals interactions within the STAT3 binding pocket. Yellow dashed lines in the three-dimensional structures indicate hydrogen-bonding interactions between the ligands and conserved active-site residues.

Docking analysis of STAT3 revealed binding energies of −5.2 and −5.0 kcal/mol for tomentosin and inuviscolide, respectively (Table 3 and Figure 7A,B). Tomentosin formed three hydrogen bonds with SER611, GLU612, and SER613, whereas inuviscolide established four hydrogen bonds involving LYS591, SER611, GLU612, and SER613 (Table 3 and Figure 7A,B). The reference inhibitor Stattic exhibited a docking score of −4.4 kcal/mol and interacted with SER611, GLU612, SER613, ARG609, and LYS591 through hydrogen bonds and salt bridges (Figure 7C). Structural studies have identified LYS591, ARG609, SER611, GLU612, SER613, SER636, and VAL637 as critical residues within the STAT3 SH2 domain that regulate STAT3 dimerization and activation [27,28]. Notably, the modeled poses of tomentosin and inuviscolide engage several of these conserved residues, closely resembling the interaction pattern reported for STAT3 inhibitors and degraders [29]. These findings support the potential of both sesquiterpenes to target the STAT3 SH2 domain and modulate STAT3-mediated oncogenic signaling, warranting experimental validation. 

### 2.10. Predicted Noncovalent Interactions of Tomentosin and Inuviscolide with the ESR1 Ligand-Binding Domain

**Figure 8:**
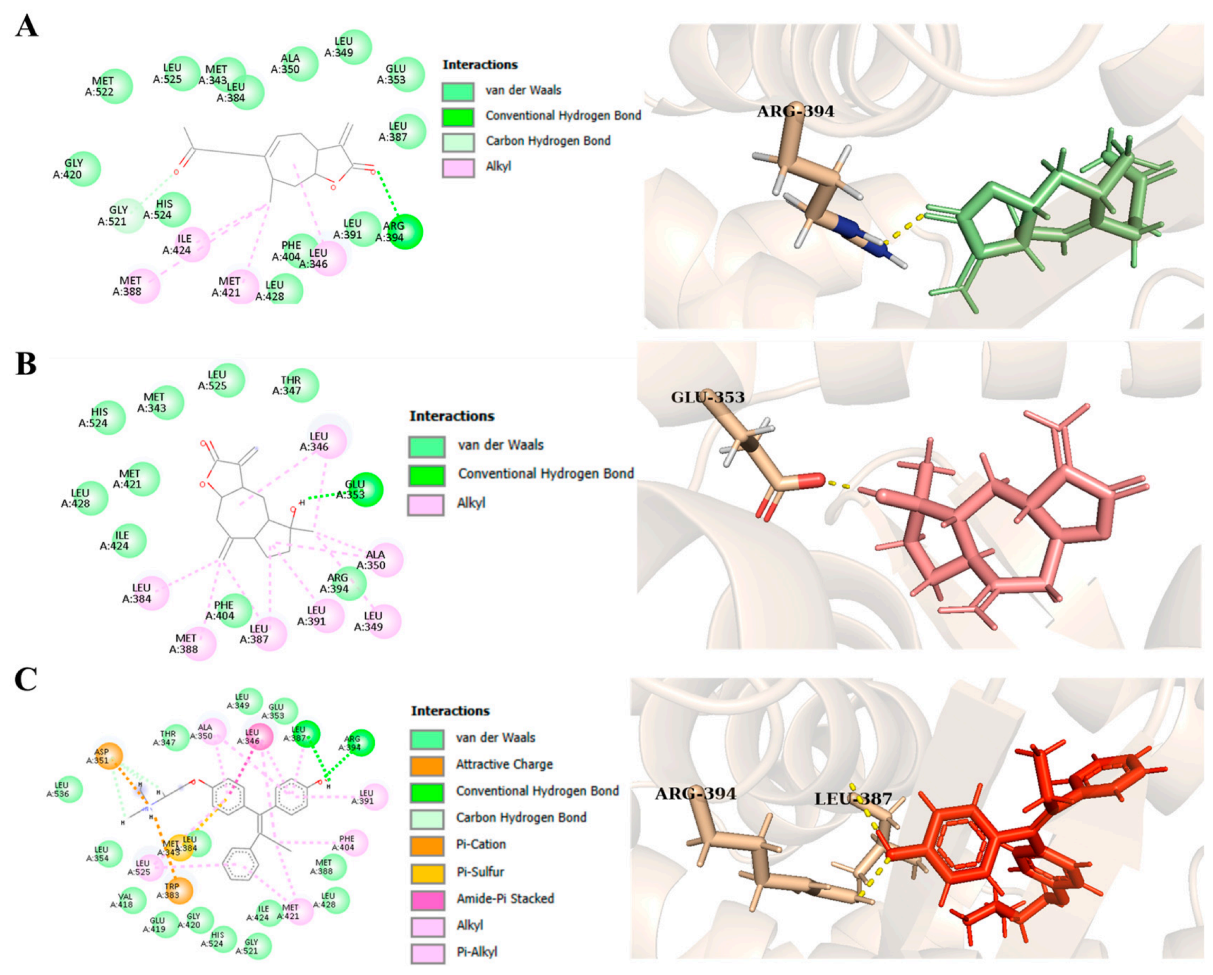
Molecular docking analysis of tomentosin and inuviscolide with the ESR1 ligand-binding domain. Two-dimensional (left) and three-dimensional (right) representations of the protein–ligand interactions between ESR1 and (**A**) tomentosin (Green), (**B**) inuviscolide (Salmon pink), and (**C**) the reference ligand 4-hydroxytamoxifen (Red). The interaction maps highlight key hydrogen bonds, hydrophobic contacts, alkyl and π-alkyl interactions, and van der Waals interac-tions that stabilize ligand binding within the ESR1 ligand-binding pocket. The three-dimensional structures illustrate the orientation of each ligand within the binding cavity, with yellow dashed lines indicating hydrogen-bonding interactions with conserved active-site residues.

To investigate their potential interactions with estrogen receptor α (ESR1), tomentosin and inuviscolide were docked against the ESR1 ligand-binding domain using 4-hydroxytamoxifen as the reference ligand. As summarized in Table 3, tomentosin exhibited the highest binding affinity, with a docking score of −7.1 kcal/mol, forming a hydrogen bond with ARG394 and extensive hydrophobic interactions involving residues such as LEU346, ALA350, MET388, LEU384, LEU387, LEU391, MET421, PHE404, and LEU525 (Figure 8A). Inuviscolide displayed a docking score of −6.8 kcal/mol and formed a hydrogen bond with GLU353, together with multiple hydrophobic interactions within the ligand-binding pocket (Figure 8B). The reference ligand 4-hydroxytamoxifen exhibited a comparable docking score of −6.8 kcal/mol and interacted with ARG394, LEU387, and several hydrophobic residues (Figure 8C). Crystallographic studies have shown that 4-hydroxytamoxifen binds ESR1 through key interactions with GLU353 and ARG394, supported by van der Waals contacts involving MET343, LEU346, THR347, ALA350, LEU384, LEU387, and LEU525 [30]. The predicted interaction profiles of tomentosin and inuviscolide show overlap with those of the reference ligand, supporting their potential ability to engage the ESR1 ligand-binding domain.

### 2.11. Tomentosin and Inuviscolide Exhibit Favorable Binding to PTGS2

**Figure 9:**
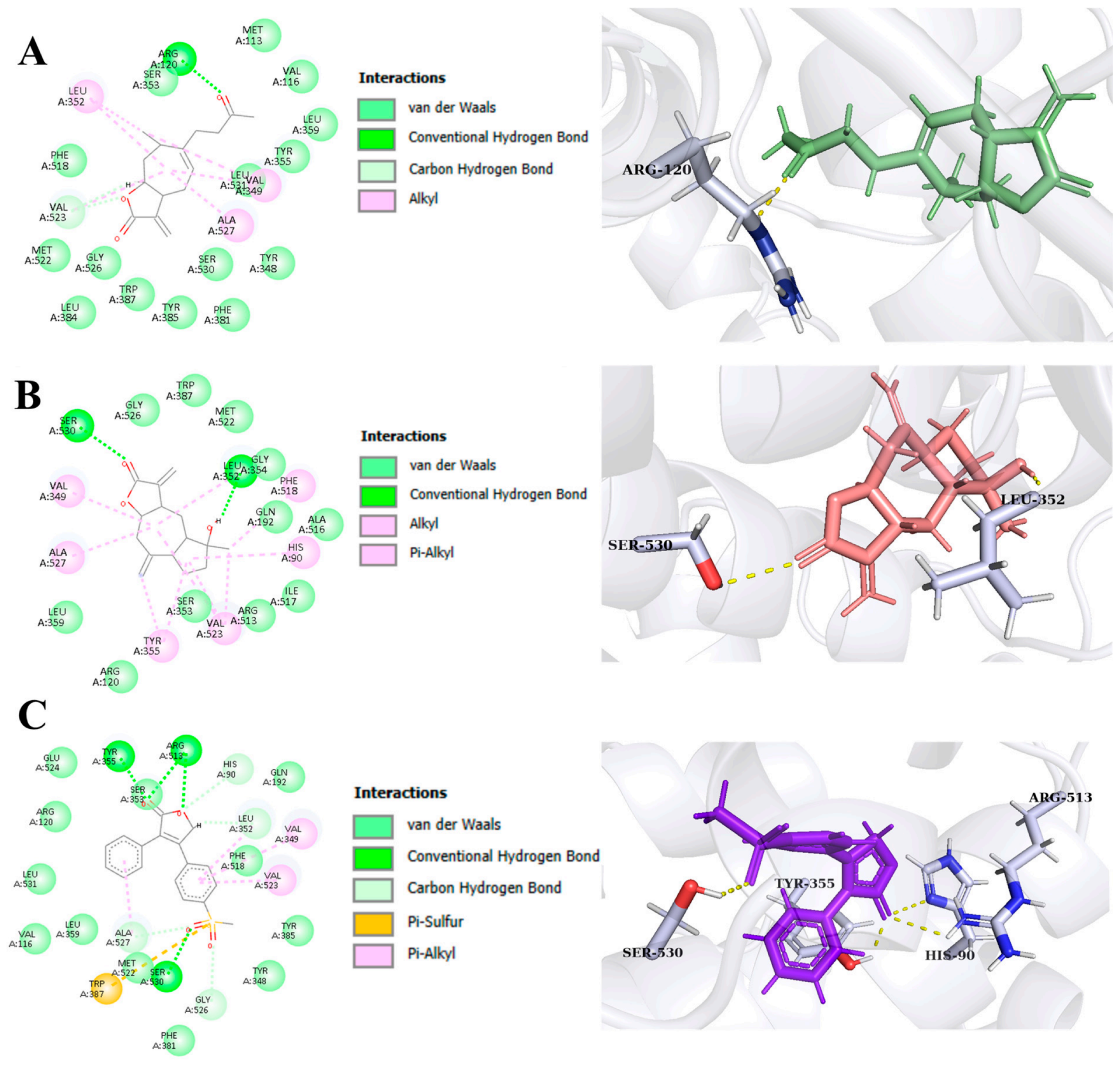
Molecular docking analysis of tomentosin and inuviscolide with the PTGS2 (COX-2) active site. Two-dimensional (left) and three-dimensional (right) representations of the protein–ligand interactions between PTGS2 and (**A**) tomentosin (Green), (**B**) inuviscolide (Salmon pink), and (**C**) the reference inhibitor rofecoxib (Purple). The interaction maps illustrate the key hydrogen bonds, hydrophobic contacts, alkyl and π-alkyl interactions, and van der Waals interactions that stabilize ligand binding within the PTGS2 catalytic pocket. The three-dimensional structures de-pict the orientation of each ligand in the active site, with yellow dashed lines indicating hydro-gen-bonding interactions with conserved catalytic residues.

To evaluate their potential as PTGS2 (COX-2) modulators, tomentosin and inuviscolide were docked against PTGS2 using rofecoxib as the reference inhibitor. As shown in Table 3, tomentosin and inuviscolide exhibited docking scores of −7.5 and −7.0 kcal/mol, respectively, comparable to that of rofecoxib (−7.2 kcal/mol). Tomentosin formed a hydrogen bond with ARG120 and established extensive hydrophobic interactions involving LEU352, VAL349, ALA527, TYR355, SER530, PHE518, TYR385, and other active-site residues (Figure 9A). Inuviscolide formed hydrogen bonds with SER530 and LEU352, together with hydrophobic interactions involving HIS90, ARG513, PHE518, VAL523, VAL349, and TYR355 (Figure 9B). The reference inhibitor rofecoxib formed hydrogen bonds with SER530, TYR355, and ARG513, along with multiple hydrophobic contacts that stabilize ligand binding within the catalytic pocket (Figure 9C). Crystallographic studies of the PTGS2–rofecoxib complex have identified HIS90, ARG513, LEU352, PHE518, ALA527, SER530, VAL349, and VAL523 as critical residues involved in selective COX-2 inhibition [31,32,33,34]. The substantial overlap between the binding residues predicted for tomentosin, inuviscolide, and rofecoxib supports the possible accommodation of both sesquiterpenes within the modeled PTGS2 active site. Overall, the molecular docking results support potential interactions of tomentosin and inuviscolide with the computationally prioritized STAT3, ESR1, and PTGS2 targets and identify key residues that may contribute to ligand recognition within the modeled binding sites. Together, the results of the network pharmacology and in vitro antiproliferative activity assays prioritize the modeled compound–target interactions for subsequent experimental investigation. In addition, these findings indicate a possible multitarget anticancer potential of *D. viscosa* sesquiterpenes and prioritize tomentosin and inuviscolide for further experimental investigation. 

Notably, tomentosin and inuviscolide contain an α-methylene-γ-lactone moiety that can act as an electrophilic Michael acceptor toward nucleophilic amino acid side chains, particularly cysteine thiols, lysine ε-amino groups, and histidine imidazole nitrogens [14,21,35,36]. This reactivity may contribute to their biological effects while also promoting broader protein modification. Inspection of the modeled ESR1 and PTGS2 complexes revealed no strongly nucleophilic residue positioned in a favorable geometry for covalent reaction. In PTGS2, the nearest candidates were SER530 and TYR355/TYR385, whose hydroxyl and phenolic groups are weak nucleophiles and were not suitably oriented for Michael addition. In ESR1, THR347 and HIS524 were located at or beyond a plausible reactive range, with the HIS524 nucleophilic nitrogen approximately 7.48 Å from the electrophilic center of tomentosin. These observations support predominantly noncovalent interactions of the modeled ESR1 and PTGS2 complexes.

In contrast, CYS687 within the STAT3 SH2 domain was pocket-facing and geometrically accessible, supporting a plausible pre-reaction orientation for thiol-Michael addition. Covalent docking using the Schrödinger Glide CovDock workflow generated feasible CYS687-linked poses for both compounds. Tomentosin yielded an estimated CovDock score of −4.09 with a sulfur-to-electrophilic-carbon separation of 2.55 Å, whereas inuviscolide yielded an estimated score of −4.79 and a separation of 2.65 Å. The modeled poses were additionally stabilized by contacts with GLU652, PHE650, TYR686, and PHE683 (Figure S19). These findings support the structural plausibility of a CYS687-directed covalent-interaction mechanism but do not experimentally establish covalent adduct formation, irreversible STAT3 engagement, or functional inhibition. Experimental confirmation through thiol-reactivity assays, protein-adduct mass spectrometry, CYS687 mutagenesis, or biochemical or cellular target-engagement studies remains necessary.

Accordingly, SwissADME and pkCSM predictions for the STAT3–sesquiterpene interactions should be interpreted cautiously because these platforms do not account for site-specific electrophilic reactivity or irreversible protein modification. 

### 2.12. Molecular Dynamics Simulations Suggest the Stable Interaction of Tomentosin and Inuviscolide with STAT3

**Figure 10:**
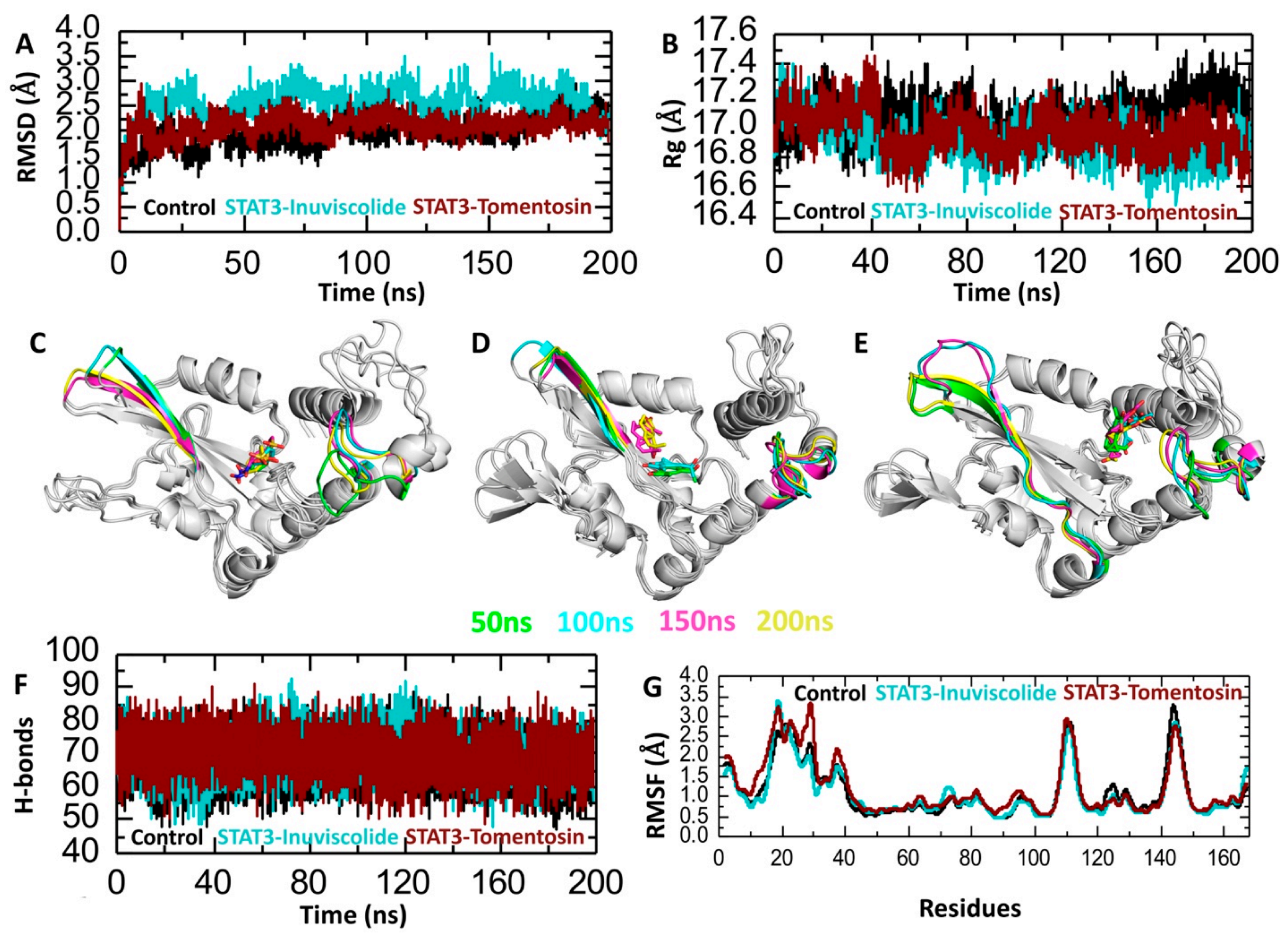
Molecular dynamics analyses of the STAT3–ligand complexes over a 200 ns simulation period. (**A**) Root mean square deviation (RMSD) profiles showing the structural stability of the STAT3–ligand complexes. (**B**) Radius of gyration (Rg) analysis illustrating the dynamic compactness of the complexes. (**C**–**E**) Superimposed ligand-binding conformations extracted at 50, 100, 150, and 200 ns. (**F**) Time-dependent total intra-protein hydrogen-bond counts in the STAT3 complexes, representing the internal protein network rather than direct protein–ligand hydrogen bonds. (**G**) Root mean square fluctuation (RMSF) analysis depicting residue-level flexibility of the STAT3–ligand complexes throughout the simulation.

To further evaluate the stability of the STAT3–ligand complexes, 200 ns molecular dynamics (MD) simulations were performed for tomentosin, inuviscolide, and the reference inhibitor Stattic. Root mean square deviation (RMSD) analysis showed that all three complexes remained structurally stable throughout the simulation without major conformational changes (Figure 10A). The Stattic–STAT3 complex exhibited the lowest fluctuations, whereas the tomentosin–STAT3 complex stabilized after an initial equilibration phase with an average RMSD of approximately 2 Å. Inuviscolide displayed slightly higher RMSD values, indicating greater conformational flexibility while maintaining overall stability [37].

Superimposition of simulation snapshots obtained at 50, 100, 150, and 200 ns showed sustained ligand accommodation within the modeled STAT3 binding pocket throughout the simulation (Figure 10C–E). Although inuviscolide underwent a slight positional shift after 150 ns, stable binding was maintained. Radius of gyration (Rg) analysis demonstrated consistent protein compactness across all systems, with tomentosin- and inuviscolide-bound complexes showing a slight decrease in Rg over time, indicative of marginally enhanced structural compactness (Figure 10B) [38,39,40].

Analysis of the total intra-protein hydrogen-bond network formed revealed that the complexes maintained approximately 70 total intra-protein hydrogen bonds during the simulation (Figure 10F). Tomentosin showed the most consistent profile, whereas inuviscolide displayed greater temporal variability. These values reflect the internal STAT3 hydrogen-bond network, not direct protein–ligand hydrogen-bond occupancy. RMSF analysis showed similar residue flexibility across systems, with the largest fluctuations in loop and terminal regions (residues 10–30, 100–120, and 140–150), while the core binding site remained stable (Figure 10G) [41,42]. 

Overall, the MD simulations suggest a sustained accommodation of tomentosin and inuviscolide within the modeled STAT3 binding pocket over the 200 ns simulation period. Tomentosin displayed a dynamic profile comparable to that of the reference inhibitor, whereas inuviscolide showed slightly greater local flexibility while maintaining its position within the modeled complex. These findings are consistent with the molecular docking and network pharmacology results and provide computational support for the proposed interactions with STAT3.

### 2.13. Molecular Dynamics Simulations Indicate the Stable Interaction of Tomentosin and Inuviscolide with ESR1

**Figure 11:**
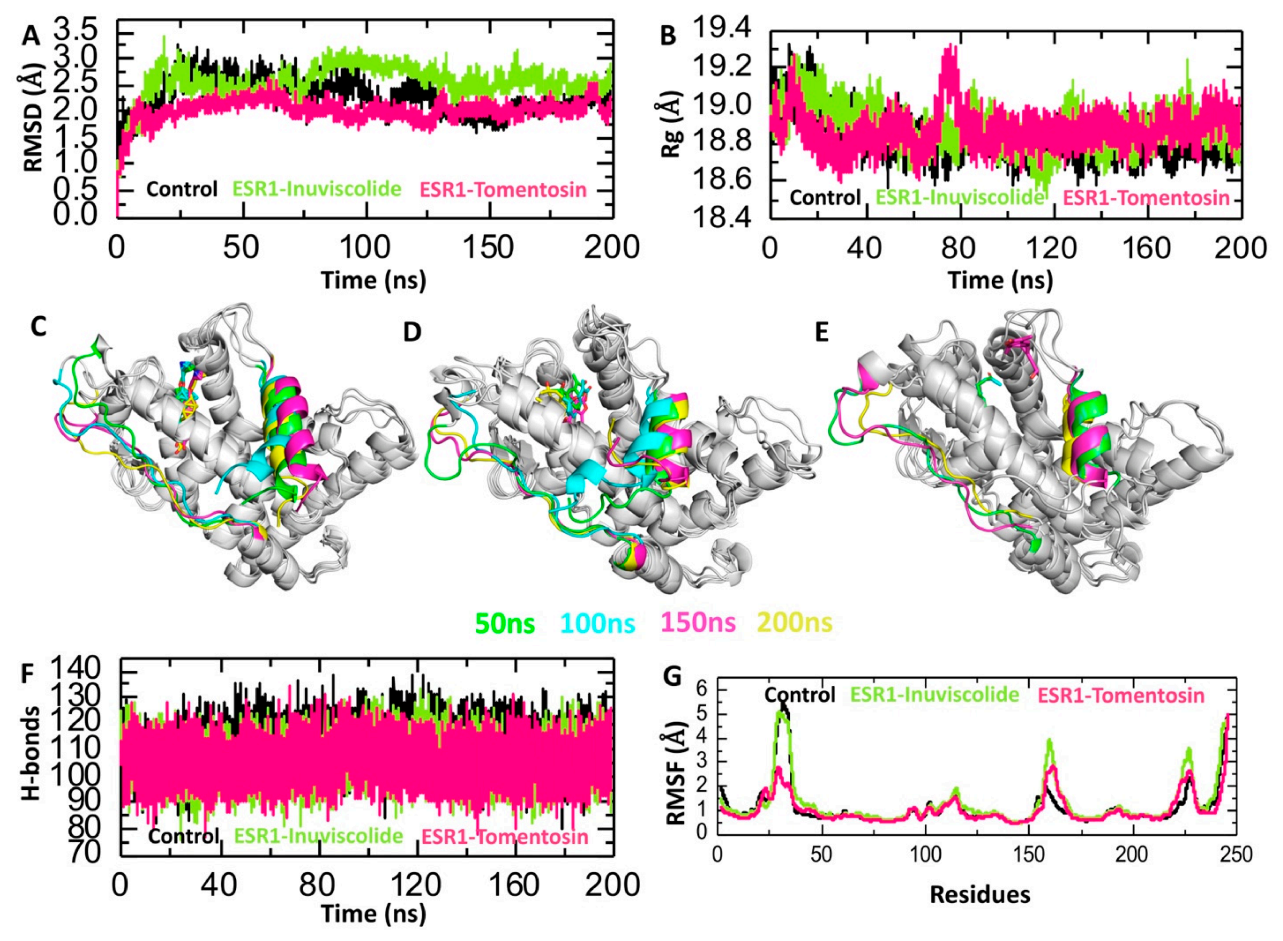
Molecular dynamics analyses of the ESR1–ligand complexes over a 200 ns simulation period. (**A**) Root mean square deviation (RMSD) profiles showing the structural stability of the ESR1–ligand complexes. (**B**) Radius of gyration (Rg) analysis illustrating the dynamic compactness of the complexes. (**C**–**E**) Superimposed ligand-binding conformations extracted at 50, 100, 150, and 200 ns. (**F**) Time-dependent total intra-protein hydrogen-bond counts in the ESR1 complexes, representing the internal protein network rather than direct protein–ligand hydrogen bonds. (**G**) Root mean square fluctuation (RMSF) analysis depicting residue-level flexibility of the ESR1–ligand complexes throughout the simulation.

To further evaluate the stability of the ESR1–ligand complexes, 200 ns molecular dynamics (MD) simulations were performed for tomentosin, inuviscolide, and the reference ligand 4-hydroxytamoxifen. Root mean square deviation (RMSD) analysis showed that all three complexes remained structurally stable throughout the simulation (Figure 11A). The control complex rapidly reached equilibrium after approximately 25 ns, whereas tomentosin exhibited the lowest and most consistent RMSD values following equilibration, with an average RMSD of approximately 2 Å. Inuviscolide displayed slightly larger RMSD fluctuations, indicating greater conformational flexibility while maintaining overall structural stability.

Superimposition of simulation snapshots obtained at 50, 100, 150, and 200 ns showed sustained ligand accommodation within the modeled ESR1 ligand-binding pocket throughout the simulation (Figure 11C–E). Tomentosin exhibited a minor positional adjustment after 150 ns, whereas inuviscolide showed modest adaptive motion consistent with its RMSD profile. Radius of gyration (Rg) analysis demonstrated stable protein compactness across all systems, with tomentosin exhibiting slightly lower Rg values and inuviscolide displaying marginally broader fluctuations (Figure 11B).

Analysis of the total intra-protein hydrogen-bond network showed that the three ESR1 complexes maintained an average of approximately 110 protein–protein hydrogen bonds during the simulation (Figure 11F). Similar intra-protein hydrogen-bond profiles were observed among the control, tomentosin, and inuviscolide systems. This analysis reflects maintenance of the internal protein hydrogen-bond network and does not quantify direct protein–ligand hydrogen-bond occupancy. Root mean square fluctuation (RMSF) analysis showed similar residue flexibility patterns for all systems, with the highest fluctuations localized to residues 25–40, 150–175, and the terminal regions. Notably, the tomentosin–ESR1 complex exhibited reduced fluctuations within the loop regions compared with the other systems (Figure 11G).

Collectively, the MD simulations indicated that both tomentosin and inuviscolide are predicted to form structurally stable complexes with ESR1 over the 200 ns simulation period. Tomentosin exhibited the greatest structural stability and compactness, whereas inuviscolide induced slightly greater conformational flexibility while preserving the overall integrity of the protein–ligand complex.

### 2.14. Molecular Dynamics Simulations Suggest the Stable Interaction of Tomentosin and Inuviscolide with PTGS2

**Figure 12:**
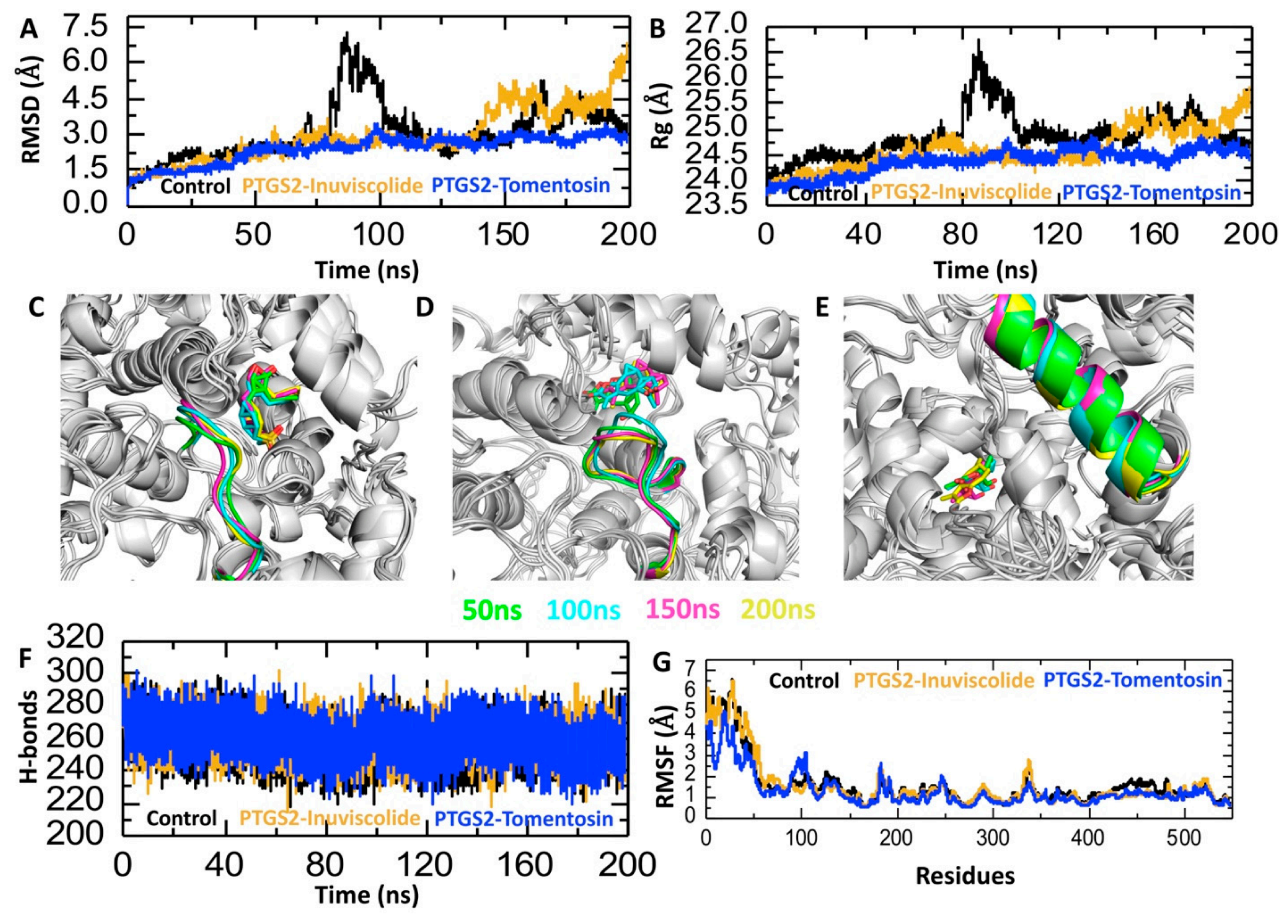
Molecular dynamics analyses of the PTGS2–ligand complexes over a 200 ns simulation period. (**A**) Root mean square deviation (RMSD) profiles showing the structural stability of the PTGS2–ligand complexes. (**B**) Radius of gyration (Rg) analysis illustrating the dynamic compactness of the complexes. (**C**–**E**) Superimposed ligand-binding conformations extracted at 50, 100, 150, and 200 ns. (**F**) Time-dependent total intra-protein hydrogen-bond counts in the PTGS2 complexes, representing the internal protein network rather than direct protein–ligand hydrogen bonds. (**G**) Root mean square fluctuation (RMSF) analysis depicting residue-level flexibility of the PTGS2–ligand complexes during the 200 ns simulation.

To further estimate the stability of the PTGS2–ligand complexes, 200 ns molecular dynamics (MD) simulations were performed for tomentosin, inuviscolide, and the reference inhibitor rofecoxib. Root mean square deviation (RMSD) analysis revealed distinct dynamic behaviors among the three systems (Figure 12A). The reference complex underwent a transient instability phase between approximately 80 and 110 ns, with RMSD values exceeding 6 Å before partially recovering. Inuviscolide exhibited moderate fluctuations followed by a gradual increase toward the end of the simulation, whereas the tomentosin–PTGS2 complex maintained the lowest and most stable RMSD profile throughout the simulation, with an average RMSD of approximately 2 Å.

Radius of gyration (Rg) analysis showed a similar trend (Figure 12B). The reference complex displayed a transient increase in Rg during its instability phase, while inuviscolide showed a gradual increase over time. In contrast, tomentosin maintained relatively constant Rg values, indicating preserved structural compactness. Superimposition of simulation snapshots obtained at 50, 100, 150, and 200 ns confirmed that all ligands remained bound within the PTGS2 active site throughout the simulation (Figure 12C–E).

Total intra-protein hydrogen-bond analysis showed that all PTGS2 complexes maintained approximately 250 to 270 protein–protein hydrogen bonds during the simulation (Figure 12F). Root mean square fluctuation (RMSF) analysis revealed similar residue flexibility profiles across all complexes, with the highest fluctuations localized primarily to the N-terminal region and reduced mobility throughout the remaining protein structure. Notably, the tomentosin–PTGS2 complex exhibited slightly lower fluctuations in several regions, suggesting enhanced local stability (Figure 12G).

Overall, the MD simulations indicated that both tomentosin and inuviscolide are predicted to form structurally stable complexes with PTGS2 over the 200 ns simulation period, supporting the initial molecular docking results. Tomentosin exhibited the most favorable dynamic profile, characterized by low RMSD values, preserved structural compactness, and reduced residue-level fluctuations, whereas inuviscolide maintained stable binding while permitting moderate conformational adaptability, supporting the molecular docking results. 

### 2.15. Post-Simulation MM/GBSA Analysis Suggests Favorable Binding Free Energies of Tomentosin and Inuviscolide

The MM/GBSA post-simulation binding free energy analysis further supported the molecular docking and MD simulation results by estimating the thermodynamic stability of the protein–ligand complexes. Both tomentosin and inuviscolide showed favorable binding toward STAT3, ESR1, and PTGS2, as indicated by negative ΔGtotal values (Table 4). Among the analyzed complexes, tomentosin exhibited the strongest binding affinity, particularly toward PTGS2 (−35.07 ± 0.3 kcal/mol), followed by ESR1 (−27.32 ± 0.3 kcal/mol) and STAT3 (−23.63 ± 0.4 kcal/mol). Inuviscolide also showed favorable binding, with ΔGtotal values of −29.88 ± 0.2, −22.99 ± 0.3, and −15.20 ± 0.2 kcal/mol for PTGS2, ESR1, and STAT3, respectively (Table 4). Energy decomposition analysis indicated that van der Waals and electrostatic interactions were major contributors to complex stabilization, whereas polar solvation energy generally opposed binding. Notably, the PTGS2 control complex displayed an unfavorable positive ΔGtotal value (+10.18 ± 0.3 kcal/mol) (Table 4), while both sesquiterpenes showed substantially stronger binding to PTGS2. Overall, the MM/GBSA results suggest that tomentosin has the most favorable predicted interaction-energy profile among the tested compounds, supporting its potential as a multitarget lead compound and its prioritization for further investigation.

## 3. Materials and Methods

### 3.1. General Experimental Procedures

SUPELCO Supelclean LC-18 SPE 10 g/60 mL cartridges (Merck, Darmstadt, Germany) were used to perform the solid phase extraction (SPE) on a SUPELCO Visiprep™ SPE Vacuum Manifold system (Merck, Darmstadt, Germany). Analytical and preparative thin layer chromatography (TLC) was performed on silica gel plates (Kieselgel 60, F_254_, 0.25 and 0.5 mm, respectively) or reverse phase (Whatman, KC18, F_254_, 0.20 mm) (Merck, Darmstadt, Germany) plates, and the compounds were visualized via exposure to UV light and/or iodine vapors and/or by spraying first with 10% H_2_SO_4_ in MeOH, and then with 5% phosphomolybdic acid in EtOH, followed by heating at 110 °C for 10 min. All the solvents employed were supplied by Sigma-Aldrich (Milan, Italy). ^1^H and ^13^C NMR nuclear magnetic resonance (NMR) spectra were recorded at 400/100 MHz on a Bruker (Karlsruhe, Germany). Electrospray ionization (ESI) mass spectra and liquid chromatography (LC-MS) analyses were performed using the LC-MS TOF system AGILENT 6230B, HPLC 1260 Infinity (Milan, Italy) in the positive modality. Optical rotations were measured on a Jasco (Tokyo, Japan) P-1010 digital polarimeter. 

### 3.2. Plant Material

*D. viscosa* plants (whole aerial parts) were collected before the blooming stage in a naturally infested area of Complesso Universitario di Monte S. Angelo, University of Naples Federico II, Naples, Italy in June 2024. The plant has been identified by Prof. Marco Masi, Department of Chemical Sciences, University of Naples Federico II, Italy, and a plant specimen is deposited in the collection of the same Department. The plant material was cleaned and dried in a fan oven at 50 °C for 2 days, then ground by using a lab mill and packaged in plastic bags under vacuum before its use.

### 3.3. Extraction and Purification of D. viscosa Metabolites

Dried aerial parts of *D. viscosa* (10 g) were macerated for 3 days at room temperature in 200 mL of MeOH/H_2_O (1:1, *v*/*v*). The resulting suspension was filtered, and the hydroalcoholic extract was lyophilized after removing MeOH under reduced pressure. To perform a solid-phase extraction (SPE) the lyophilized material was suspended in 60 mL of distilled water and loaded onto an SPE column, which was previously conditioned with 60 mL of MeOH and equilibrated with 120 mL of distilled water. The purification yielded four fractions (F_1_–F_4_) obtained via a stepwise elution with H_2_O (60 mL, F_1_), MeOH/H_2_O 7:3, *v*/*v* (120 mL, F_2_), CH_3_CN/H_2_O 7:3, *v*/*v* (120 mL, F_3_), and CH_3_CN (120 mL, F_4_), respectively. Successively, the organic solvents were removed under reduced pressure, and the eventual residual water phases were lyophilized, yielding 140.3 mg (F_1_), 98.1 mg (F_2_), 34.0 mg (F_3_), and 3.2 mg (F_4_), respectively. The residue of F_2_ was purified using two subsequent steps of preparative TLC eluted with CHCl_3_/*i*-PrOH (98:2, *v*/*v*) and TLC in reverse phase eluted with CH_3_CN/H_2_O (7:3, *v*/*v*), yielding tomentosin (55.1 mg), 11α,13-dihydrotomentosin (8.1 mg), inuviscolide (1.8 mg), and *α*-costic acid (15.3 mg) as oily compounds. The residue of F_3_ was purified by preparative TLC eluted with EtOAc/*n*-hexane (1/1, *v*/*v*), yielding a further amount of *α*-costic acid (25.1 mg).

### 3.4. Cytotoxicity of Selected Sesquiterpenes Against A549 and HCT116 Cells

The human lung adenocarcinoma (A549) colorectal carcinoma (HCT116) and non-malignant human neonatal dermal fibroblasts (HDFn) were obtained from the American Type Culture Collection (ATCC; Manassas, VA, USA). Cells were cultured in DMEM Medium (Gibco, Thermo Fisher Scientific, Waltham, MA, USA) supplemented with 10% fetal bovine serum (FBS; Gibco), 100 U/mL penicillin, and 0.1 mg/mL streptomycin. Cells were maintained in a humidified incubator at 37 °C with 5% CO_2_. Media were refreshed every 2–3 days, and cells were subcultured at 80–90% confluence using 0.05% trypsin-EDTA. Tomentosin, 11α,13-dihydrotomentosin, inuviscolide, and *α*-costic acid were dissolved in DMSO to prepare stock solutions, which were further diluted to working concentrations in DMEM. Cell viability was assessed using the Alamar Blue assay (Invitrogen, Thermo Fisher Scientific) according to the manufacturer’s instructions. A total of 5.0 × 10^3^ cells per well were seeded in 96-well plates and allowed to adhere for 24 h. Cells were then treated with tomentosin, 11α,13-dihydrotomentosin, inuviscolide, and *α*-costic acid at final concentrations between 2.5 and 300 µM or DMSO, at a concentration equivalent to that present in the highest treatment concentration (less than 0.5% DMSO), as a vehicle control. In our routine antiproliferative screening workflow, treatment effects are evaluated after 24, 48, and 72 h. Based on previous studies in which concentration-dependent effects were most clearly captured after 48 h [43,44], a 48 h exposure period was selected a priori and applied uniformly to all cell lines. After 48 h of treatment, 10% (*v*/*v*) Alamar Blue reagent was added to each well and incubated for 4 h at 37 °C in the dark. Fluorescence was measured at 560 nm excitation and 590 nm emission using a TECAN Infinite M200 microplate reader (Männedorf, Switzerland). Cell viability was expressed as a percentage relative to the DMSO-treated vehicle control group. The control viability was set at 100%, and cell viability was calculated using the formula % Viability = (Fluorescence of treated cells/Fluorescence of control cells) × 100. IC_50_ values were determined from the concentration–response curves using GraphPad Prism Ver. 10. The Selectivity Index was calculated as SI = IC_50_ in HDFn cells/IC_50_ in the corresponding cancer cell line. All treatments were performed in technical triplicate wells, and experiments were repeated independently three times (*n* = 3). 

### 3.5. Statistical Analysis

All in vitro experiments were performed using three independent biological replicates (*n* = 3), with each biological replicate analyzed in triplicate technical replicates. Statistical analyses were conducted using GraphPad Prism version 10 (GraphPad Software, Boston, MA, USA). Data are presented as the mean ± standard deviation (SD). Statistical differences among experimental groups were evaluated by one-way analysis of variance (ANOVA), followed by Tukey’s multiple comparisons post hoc test. A *p* value of < 0.05 was considered statistically significant.

The half-maximal inhibitory concentration (IC_50_) values were estimated by nonlinear regression analysis using a logistic dose–response model in GraphPad Prism. Dose–response curves were generated by plotting the percentage of cell viability relative to the vehicle control against the logarithm of the treatment concentrations, and IC_50_ values were calculated from the fitted curves.

### 3.6. Evaluation of Physicochemical, Drug-Likeness, and ADMET Properties

The physicochemical properties, drug-likeness, and pharmacokinetic profiles of tomentosin, 11*α*,13-dihydrotomentosin, inuviscolide, and *α*-costic acid were evaluated using in silico approaches. ADMET analysis is widely used during early-stage drug discovery to evaluate the pharmacokinetic behavior, safety, and drug-development potential of bioactive compounds. The SMILES notations of the compounds were retrieved from PubChem (https://pubchem.ncbi.nlm.nih.gov/, accessed on 4 June 2026) and submitted to the SwissADME web server [45] to predict their physicochemical characteristics and drug-likeness properties. Parameters including molecular weight, lipophilicity (LogP), topological polar surface area (TPSA), molar refractivity (MR), aqueous solubility, bioavailability score, and compliance with Lipinski’s Rule of Five (RO5) were assessed. Furthermore, the absorption, distribution, metabolism, excretion, and toxicity (ADMET) profiles of the compounds were predicted using the pkCSM pharmacokinetic web server (https://biosig.lab.uq.edu.au/pkcsm/, accessed on 4 June 2026) [46]. 

### 3.7. Density Functional Theory (DFT) Analysis of D. viscosa Sesquiterpenes

In order to evaluate electronic characteristics and chemical reactivity of the four sesquiterpenes, density functional theory (DFT) calculations were performed. All computations were conducted using the Jaguar module within the Maestro Schrodinger Suite (Schrodinger LLC, New York, NY, USA). First, the three-dimensional structures of the selected compounds were submitted to full geometry optimization to obtain their most energetically favorable conformations. Subsequently, the optimized structures were analyzed using the B3LYP hybrid exchange-correlation functional in combination with the 6-31G* basis set. Frontier molecular orbital (FMO) analysis was carried out to determine the energies and spatial distributions of the highest occupied molecular orbital (HOMO) and lowest unoccupied molecular orbital (LUMO). The HOMO–LUMO energy gap (ΔE) was then calculated to assess molecular stability, electron-transfer potential, and overall chemical reactivity. Furthermore, several global quantum chemical descriptors were derived, including ionization potential (IP), electron affinity (EA), electronegativity (χ), chemical potential (μ), chemical hardness (η), chemical softness (σ), electrophilicity index (ω), dipole moment, and electron density area (EDA), providing comprehensive insights into the electronic behavior and interaction potential of the investigated phytocompounds.

### 3.8. Prediction of D. viscosa Sesquiterpene Protein Targets and Disease Targets

The SMILES of tomentosin, 11α,13-dihydrotomentosin, inuviscolide, and α-costic acid, obtained from PubChem, were imported into the SwissTargetPrediction network database (http://www.swisstargetprediction.ch/, accessed 9 April 2026) [47] and Way2Drug PASS Targets (https://way2drug.com/passtargets/, accessed 9 April 2026) [48]. Further, the targets from both databases were merged, and duplicates were removed to obtain a final list of targets. Having tested the in vitro activity of these compounds in lung and colorectal cancer cell lines, we aimed to identify the disease-specific target genes for these two cancer types. The targets for both lung and colorectal cancer were retrieved from the Genecards database (https://www.genecards.org/), NCBI (https://www.ncbi.nlm.nih.gov/gene/ accessed on 9 April 2026), and MalaCards (https://www.malacards.org/) by independently searching for the terms “lung cancer” and “colorectal cancer” as keywords. Genes from the three databases were combined, and duplicate gene entries were removed.

### 3.9. Overlapping of Compound–Disease Target Genes

Overlapping targets between the cancer types and compound targets were identified using the Venny 2.0 tool (https://bioinfogp.cnb.csic.es/tools/venny/index2.0.2.html, accessed 9 April 2026). These intersecting genes were selected for constructing the protein–protein interaction (PPI) network and for further analysis. 

### 3.10. Construction of the Protein–Protein Interaction (PPI) Network 

For constructing the PPI network, the overlapping targets between compound targets and cancer-related targets, identified using the Venn tool, were input into STRING version 12.0 (http://www.string-db.org/, accessed 9 April 2026) [49]. The PPI network was then created by filtering for *Homo sapiens* and applying a highest confidence of 0.9 to ensure relevance and reliability. Data from the STRING database was downloaded in TSV format and imported into Cytoscape version 3.10.3 (http://www.cytoscape.org/, accessed 9 April 2026) for further analysis [50]. Cytoscape reveals topology parameters of nodes in the network, including betweenness centrality (BC), closeness centrality (CC), and degree centrality (DC) values [51]. Using the degree score, the Cytohubba plug-in was utilized to identify the top 10 hub genes in the network [52]. Among the four compounds, tomentosin and inuviscolide exhibited the most potent in vitro antiproliferative activity. Accordingly, we have selected the top three hub genes common between the tomentosin and inuviscolide targets for further in silico analyses. 

### 3.11. Gene Ontology and KEGG Pathway Enrichment Analysis

GO enrichment analysis and KEGG pathway enrichment analysis were performed separately for the complete sets of 322 lung cancer-associated and 337 colorectal cancer-associated compound–disease targets using the ShinyGO web server (http://bioinformatics.sdstate.edu/go/, accessed 9 April 2026). KEGG pathway analysis provides an understanding of the functional role of the compounds and their involvement in cancer-related pathways by assessing BPs, CCs, MFs, and associated signaling pathways [53,54,55]. The analysis was performed using an FDR (False Discovery Rate) cut-off of 0.05 and identified the top diseases, functions, and enriched pathways associated with the target genes. 

### 3.12. Molecular Docking of D. viscosa Sesquiterpenes with the Selected Hub Proteins

#### 3.12.1. Noncovalent Molecular Docking

To investigate the potential molecular interactions underlying the anticancer activity of *D. viscosa* sesquiterpenes, molecular docking studies were performed using AutoDock Vina v1.2.0 [56,57]. A grid-based targeted docking approach was employed to predict the binding affinities and interaction modes of the selected compounds with key hub proteins implicated in colorectal and lung cancer pathogenesis. The three-dimensional crystal structures of the target proteins, including signal transducer and activator of transcription 3 (STAT3; PDB ID: 6NUQ), prostaglandin-endoperoxide synthase 2 (PTGS2/COX-2; PDB ID: 5KIR), and estrogen receptor alpha (ESR1; PDB ID: 3ERT), were retrieved from the Protein Data Bank (PDB) [29,30,31,58]. Protein structures were prepared by removing crystallographic water molecules and co-crystallized ligands, followed by the addition of polar hydrogen atoms and Kollman charges to generate docking-ready receptor models. 

Based on the in vitro antiproliferative screening, tomentosin and inuviscolide were selected for docking and molecular simulation studies because they showed the best antiproliferative activity among the four compounds tested against HCT116 and A549 cells. The three-dimensional (3D) structures of tomentosin (PubChem CID: 155173), inuviscolide (PubChem CID: 176489), and the reference inhibitors Stattic (PubChem CID: 2779853), rofecoxib (PubChem CID: 5090), and 4-hydroxytamoxifen (PubChem CID: 449459) were retrieved from the PubChem database in SDF format. Ligand preparation was performed using AutoDock Tools (ADT, version 1.5.7), where polar hydrogen atoms and Gasteiger partial charges were added, non-polar hydrogens were merged, and rotatable bonds were assigned to allow conformational flexibility during docking. Prior to docking, the ligand structures were energy-minimized using the MMFF94 force field implemented in Open Babel (version 3.1.1). The prepared ligands were subsequently converted to the PDBQT format required for molecular docking with AutoDock Vina. 

Docking grids were centered on the co-crystallized inhibitor-binding pockets of the target proteins in order to ensure accurate prediction of ligand binding within the active sites. In particular, the grid center coordinates for STAT3 were set at X = 13.711, Y = 54.024, and Z = −0.083 with a grid spacing of 0.375 Å; the grid center coordinates for PTGS2 (COX-2) were X = 29.54, Y = 29.74, and Z = 27.37, and the grid center coordinates for ESR1 were X = 30.282, Y = −2.033, and Z = 24.207 with a grid spacing of 0.375 Å. The exhaustiveness parameter was set to 32 to enhance the accuracy and reliability of the docking predictions. Docking simulations were performed using AutoDock Vina v1.2.0, and the binding affinities of tomentosin and inuviscolide were compared with those of the corresponding reference inhibitors. For each ligand, ten binding poses were generated, and the optimal conformation was selected based on the lowest binding free energy and the most favorable interaction pattern within the target binding site. Protein–ligand complexes were subsequently visualized and analyzed using PyMOL Molecular Graphics System (Version 3.0, Schrödinger, LLC, New York, NY, USA) and BIOVIA Discovery Studio Visualizer to characterize the binding modes and key intermolecular interactions. 

#### 3.12.2. Covalent Molecular Docking

Low-energy noncovalent poses of tomentosin and inuviscolide with STAT3, ESR1, and PTGS2 were inspected for accessible nucleophilic residues positioned near the α-methylene-γ-lactone electrophile. No favorable reactive nucleophile was identified in the modeled ESR1 or PTGS2 complexes, whereas CYS687 in the STAT3 SH2 domain was pocket-facing and geometrically accessible. It was therefore selected for covalent docking.

Covalent docking was performed using Glide CovDock module in Schrödinger Maestro, Release 2022-4. STAT3 (PDB ID: 6NUQ) was prepared with the Protein Preparation Wizard by assigning bond orders, adding hydrogen atoms, creating disulfide bonds where appropriate, and assigning protonation states at pH 7.4. CYS687 in chain A was prepared in the reactive state required for Michael addition and designated as the receptor attachment residue. Tomentosin and inuviscolide were prepared using LigPrep. Ionization and tautomeric states were generated with Epik at pH 7.4 ± 1.0, while the specified stereochemistry and intact α-methylene-γ-lactone moiety were retained. The lowest-energy prepared state of each ligand was used. The grid was centered on the STAT3 SH2 binding pocket. CovDock was run in Pose Prediction mode using the predefined Michael-addition reaction, with CYS687 as the nucleophile and the exocyclic β-carbon as the electrophilic center, with no leaving group. The workflow included Glide pose generation, covalent bond formation, Prime refinement, and final scoring.

Poses were ranked by CovDock score, and the best-ranked complex for each ligand was selected. Glide GScore, Glide Emodel, and Prime energy values were recorded. Selected complexes were exported in PDB format and examined using PyMOL. Sulfur-to-electrophilic-carbon separations were calculated using a custom Python 3.12.7 script, and three-dimensional representations were generated with Matplotlib 3.9.2

### 3.13. Molecular Dynamics Simulation of Selected Ligand–Target Complexes 

Molecular docking was followed by molecular dynamics (MD) simulations to evaluate the stability and conformational behavior of the modeled protein–ligand complexes of tomentosin and inuviscolide with the computationally prioritized candidate targets. Coordinate and topology files were generated using the tLEaP module of AMBER21 [59,60]. Proteins were parameterized with the ff19SB force field, whereas ligands were parameterized using GAFF2 [61]. Ligand partial charges were assigned using the AM1-BCC method through Antechamber, and missing parameters were generated with Parmchk2 [61]. Each complex was solvated in a truncated octahedral OPC water box with a minimum solute-to-boundary distance of 10 Å, and appropriate counterions were added for charge neutralization [61]. 

Energy minimization was performed in two stages. Solvent molecules and counterions were first minimized for 18,000 steps, comprising 9000 steepest-descent and 9000 conjugate-gradient steps, while the protein–ligand complex was restrained with a force constant of 10 kcal mol^−1^ Å^−2^. The entire system was then minimized without restraints for 10,000 steps, comprising 5000 steepest-descent and 5000 conjugate-gradient steps.

The minimized systems were heated from 0 to 300 K over 100 ps under the NVT ensemble using a Langevin thermostat with a collision frequency of 1.0 ps^−1^. During heating, the protein backbone was restrained with a force constant of 10 kcal mol^−1^ Å^−2^. The systems were then equilibrated for 500 ps under the NPT ensemble at 300 K and 1 atm, with positional restraints gradually removed to permit density relaxation. Pressure was controlled using the Berendsen barostat with a relaxation time of 2.0 ps. Long-range electrostatic interactions were treated using the Particle Mesh Ewald method, and a 10 Å cutoff was applied to short-range nonbonded interactions [62]. Bonds involving hydrogen atoms were constrained using SHAKE, allowing a 2 fs integration timestep [61]. Each system was subsequently subjected to a 200 ns NPT production simulation at 300 K and 1 atm using the Langevin thermostat with a collision frequency of 1.0 ps^−1^ and the Berendsen barostat with ntp = 1 and a relaxation time of 2.0 ps.

### 3.14. Post-Simulation Analysis of the Compound–Target Complexes

The trajectories obtained from the production phase of the simulations were analyzed via the CPPTRAJ and PTRAJ modules of AMBER [63]. Structural and dynamic parameters, including root mean square deviation (RMSD), root mean square fluctuation (RMSF), radius of gyration (Rg), and total intra-protein hydrogen-bond profiles, were computed for each system [64,65]. The hydrogen-bond analysis quantified hydrogen bonds formed within the protein structure and was used to assess preservation of the internal protein hydrogen-bond network. It did not quantify direct protein–ligand hydrogen-bond occupancy.

The RMSD, which quantifies the average positional deviation between the reference and superimposed structures, was calculated according to the equation

(1) $$RMSD=\sqrt{\frac{\sum d^{2}i=1}{N}}$$

where *i* is the index of individual atoms, *di* is the distance between the position of atom *i* at a given time and its reference position, and *N* is the total number of atoms considered in the calculation.

RMSF was calculated according to the equation

(2) $$RMSF=\sqrt{\frac{3B}{8\pi^{2}}}$$

where *B* is the B-factor (Debye–Waller or temperature factor) calculated as

(3) $$B=\frac{8\pi^{2}}{3}\left\langle \Delta r^{2}\right\rangle$$

where *Δr* is the atomic position, and *Δr^2^* represents the mean square displacement of an atom from its average position.

Rg was calculated using

(4) $$r_{Rg}^{2}=\frac{\sum_{i=1}^{N}m_{i}{\left(r_{i}-r_{CM}\right)}^{2}}{\sum_{i=1}^{N}m_{i}}$$

where *r_Rg_* = Radius of gyration (Å), mi is the mass of atom I, *r_i_* is the position vector of atom I, and rCM is the position vector of the center of mass of the molecule.

### 3.15. Binding Free Energies Calculation of Compound–Targets Complexes 

To further validate the stability and reliability of the protein–ligand interactions identified through molecular docking, Molecular Mechanics/Generalized Born Surface Area (MM/GBSA) calculations were performed on the selected complexes. Conventional docking methods estimate binding affinity primarily based on static scoring functions, while the MM/GBSA approach provides a more comprehensive assessment of ligand binding by incorporating multiple energetic factors involved in complex formation. These include van der Waals interactions, electrostatic forces, polar solvation energy, and nonpolar solvation effects, allowing for a more realistic estimate of binding free energy under physiological conditions. The binding free energies of the compound–targets complexes were calculated according to the standard MM/GBSA equations, as described below [66,67].

(5) $${∆G}_{bind}=G_{\left(complex,\;solvated\right)}-G_{\left(ligand,\;solvated\right)}-G_{\left(protein,\;solvated\right)}$$

Individual contribution to the complexes was calculated as

(6) $$G=E_{Molecular\;Mechanics}+G_{solvated}-TS$$

(7) $${∆G}_{bind}={∆E}_{Molecular\;Mechanics}+{∆G}_{solvated}-∆TS={∆G}_{vaccum}+{∆G}_{solvated}$$

(8) $${∆E}_{Molecular\;Mechanics}={∆E}_{int}+{∆E}_{electrostatic}+{∆E}_{vdW}$$

(9) $$∆G_{solvated}=∆G_{Generalized\;born}+∆G_{surface\;area}$$

(10) $$∆G_{surface\;area}=\gamma .SASA+b$$

(11) $$∆G_{vaccum}={∆E}_{Molecular\;Mechanics}-T∆S$$

## 4. Conclusions

Among the four *D. viscosa* sesquiterpenes evaluated, tomentosin and inuviscolide showed the greatest antiproliferative activity against HCT116 colorectal and A549 lung cancer cells, with IC_50_ values in the moderate micromolar range. Selectivity assessment in HDFn cells revealed compound- and cancer-type-dependent responses, with inuviscolide showing the highest selectivity toward HCT116 cells. DFT and ADMET predictions characterized the electronic, physicochemical, pharmacokinetic, and toxicity-related properties of the compounds, supporting the prioritization of tomentosin and inuviscolide as natural-product scaffolds rather than optimized anticancer candidates.

Network pharmacology and PPI analyses prioritized STAT3, ESR1, and PTGS2 as computational candidate targets shared between the two cancer types, while enrichment analyses indicated processes related to tumor proliferation, inflammation, immune regulation, and cellular stress. These findings should be interpreted within the broader polypharmacological profile of *D. viscosa* sesquiterpenes and not as evidence of single-target activity or a confirmed mechanism of action.

Noncovalent docking, 200 ns molecular dynamics simulations, and MM/GBSA calculations suggested plausible modeled interactions of tomentosin and inuviscolide with the prioritized proteins, with tomentosin showing the most favorable predicted binding profile. Covalent docking also identified structurally feasible CYS687-linked poses with STAT3, although this remains a computational outcome requiring experimental confirmation.

Collectively, the findings prioritize tomentosin and inuviscolide as *D. viscosa*-derived sesquiterpene scaffolds for further pharmacological benchmarking, structural optimization, selectivity assessment, and direct mechanistic validation 

Future studies should include clinically relevant reference drugs tested under identical conditions, complementary functional assays, and direct biochemical and cellular target validation. Thiol-reactivity assays, protein-adduct mass spectrometry, CYS687 mutagenesis, and appropriate loss-of-function approaches will be needed to examine the predicted covalent interaction with STAT3. Broader testing across molecular diverse cancer models will also be important to assess the reproducibility, cancer-type specificity, and biological relevance of the observed activity. Further work should address structural optimization, pharmacological benchmarking, pharmacokinetic and metabolic characterization, and preclinical efficacy and safety assessment to clarify the biological significance of tomentosin and inuviscolide as natural-product scaffolds with moderate antiproliferative activity.

## Figures and Tables

**Table 1:** In vitro antiproliferative activity IC_50_ and selectivity index (SI) of *D. viscosa* sesquiterpenes against HCT-116, A549, and HDFn cells after 48 h of exposure.

Compound	A549 IC_50_ (µM)	A549 SI	HCT-116 IC_50_ (µM)	HCT-116 SI	HDFn IC_50_ (µM)
Tomentosin	23.20 ± 1.26	5.23	17.82 ± 3.72	6.81	121.28 ± 8.36
11*α*,13-Dihydrotomentosin	149.27 ± 7.21	1.19	86.84 ± 5.61	2.04	177.14 ± 9.58
Inuviscolide	28.63 ± 5.90	5.50	15.72 ± 3.81	10.02	157.53 ± 12.36
*α*-Costic acid	179.31 ± 8.90	0.98	164.09 ± 7.62	1.08	176.54 ± 5.32

**Table 2:** Physicochemical, drug-likeness, and absorption, distribution, metabolism, excretion, and toxicity (ADMET) prediction of *D. viscosa* sesquiterpenes.

Compound Name	*α*-Costic Acid	Tomentosin	11*α*,13-Dihydrotomentosin	Inuviscolide
Physicochemical properties				
MW (g/mol)	234.33	248.32	250.33	248.32
Hydrogen bond acceptors	2	3	3	3
HBD (hydrogen bond)	1	0	0	1
MR (molar refractivity)	70.55	70.53	71	69.41
TPSA (Å2)	37.3	43.37	43.37	46.53
Consensus Log P	3.32	2.53	2.55	2.32
ESOL class	Soluble	Soluble	Soluble	Soluble
Lipinski rule	0	0	0	0
Bioavailability score	0.85	0.55	0.55	0.55
Absorption				
Caco2 permeability	1.592	1.322	1.326	1.279
Intestinal absorption	94.037	98.631	98.484	96.853
Skin permeability	−2.701	−2.706	−2.696	−3.613
Distribution				
VD (human) (L/kg)	−0.482	0.139	0.142	0.229
BBB permeability	0.385	0.267	0.268	0.081
CNS permeability	−2.264	−2.606	−2.606	−2.89
Metabolism				
CYP2C19 inhibitor	No	No	No	No
CYP2C9 inhibitor	No	No	No	No
CYP2D6 inhibitor	No	No	No	No
CYP3A4 inhibitor	No	No	No	No
Excretion				
Total clearance	1.166	1.276	1.172	1.121
Renal OCT2 substrate	No	No	No	No
Toxicity				
AMES toxicity	No	No	No	No
Hepatotoxicity	No	No	No	No

**Table 3:** Docking scores and interactions of tomentosin, inuviscolide, and comparative reference ligands with STAT3, ESR1, and PTGS2.

Target	Compound Name	Docking Score (kcal/mol)	Hydrogen Bond/Salt Bridge	Hydrophobic Interaction
STAT3	Tomentosin	−5.2	SER611, GLU612, SER613	PRO639, THR620, SER636, GLU638, VAL637
	Inuviscolide	−5	LYS591 SER613, SER611, GLU612	PRO639, THR620, ARG609, GLN635, SER636
	Stattic (Control)	−4.4	SER611, GLU612, SER613, ARG609, LYS591	PRO639, SER636, GLU638, VAL637
ESR1	Tomentosin	−7.1	ARG394	ILE424, MET388, MET421, LEU346, GLU353, LEU387, ALA350, LEU384, MET343, LEU525, MET522, GLY420, LEU428, PHE404, LEU391
	Inuviscolide	−6.8	GLU353	LEU346, ALA350, MET388, LEU349, LEU384, LEU391, THR347, HIS524, ILE424, MET421, GLU353, LEU387, MET343, LEU525, LEU428, PHE404
	4-hydroxytamoxifen (control)	−6.8	LEU387, ARG394	LEU391, PHE404, MET421, LEU525, ALA350, LEU346, GLU353, LEU349, THR347, LEU536, LEU354, VAL418, GLU419, GLY420, HIS524, GLY521, LEU428, MET388, ILE424
PTGS2	Tomentosin	−7.5	ARG120	LEU352, VAL349, ALA527, MET113, VAL116, LEU359, TYR355, LEU531, SER530, TYR348, SER530, TYR348, PHE 381, TYR385, PHE381, TRP387, GLY526, MET522, LEU384, PHE518
	Inuviscolide	−7	SER530, LEU352	PHE518, HIS90, VAL523, VAL349, TYR355, TRP387, GLY526, MET522, GLY354, ALA516, ILE517, ARG513, SER353, ARG120, LEU359,
	Rofecoxib (control)	−7.2	SER530, TYR355, ARG513	VAL349, VAL523, GLU524, ARG120, LEU531, VAL116, LEU359, ALA527, MET522, GLY526, TYR348, TYR385, PHE518, GLN192, HIS90, LEU352

**Table 4:** MM/GBSA post-simulation binding free energy components of selected compound–target complexes.

Parameters	ΔEvdw	ΔEele	EGB	ESURF	Delta G Gas	Delta G Solv	∆G Total
Control-STAT3	−20.71 ± 0.3	−62.83 ± 1.1	72.84 ± 0.9	−2.97 ± 0.0	−83.54 ± 1.0	69.86 ± 0.9	−13.68 ± 0.3
Inuviscolide-STAT3	−18.72 ± 0.2	−13.19 ± 0.5	19.08 ± 0.5	−2.37 ± 0.0	−31.91 ± 0.6	16.71 ± 0.5	−15.20 ± 0.2
Tomentosin-STAT3	−21.23 ± 0.3	−153.90 ± 2.1	154.29 ± 1.8	−2.79 ± 0.0	−175.13 ± 1.9	151.50 ± 1.8	−23.63 ± 0.4
Control-ESR1	−20.40 ± 0.4	190.20 ± 1.3	−188.07 ± 1.3	−2.90 ± 0.0	183.81 ± 1.5	−190.97 ± 1.3	−21.16 ± 0.4
Inuviscolide-ESR1	−32.09 ± 0.2	142.91 ± 1.2	−129.66 ± 1.1	−4.14 ± 0.0	110.82 ± 1.2	−133.81 ± 1.1	−22.99 ± 0.3
Tomentosin-ESR1	−42.86 ± 0.4	123.78 ± 1.5	−101.67 ± 1.6	−6.56 ± 0.0	60.91 ± 1.7	−108.23 ± 1.5	−27.32 ± 0.3
Control-PTGS2	−35.99 ± 0.2	−54.41 ± 0.8	105.79 ± 0.7	−5.21 ± 0.0	−90.40 ± 0.8	100.58 ± 0.7	10.18 ± 0.3
Inuviscolide- PTGS2	−33.95 ± 0.2	−236.50 ± 1.5	244.75 ± 1.4	−4.18 ± 0.0	−270.45 ± 1.5	240.57 ± 1.4	−29.88 ± 0.2
Tomentosin- PTGS2	−32.96 ± 0.2	−212.43 ± 1.0	215.21 ± 0.9	−4.89 ± 0.0	−245.40 ± 1.0	210.33 ± 0.9	−35.07 ± 0.3

## Data Availability

The data that support the findings of this study are available from the corresponding author upon reasonable request.

## Supplementary Materials

The following supporting information can be downloaded at https://www.mdpi.com/article/10.3390/ijms27157064/s1.

## References

[1] Sladonja B., Poljuha D., Krapac M., Uzelac M., Mikulic-Petkovsek M. (2021). *Dittrichia viscosa*: Native-Non Native Invader. Diversity.

[2] Jerada R., Er-Rakibi A., Cherkani Hassani A., Benzeid H., El Ouardi A., Harhar H., Goh B.H., Yow Y.Y., Ser H.L., Bouyahya A. (2024). A Comprehensive Review on Ethnomedicinal Uses, Phytochemistry, Toxicology, and Pharmacological Activities of *Dittrichia viscosa* (L.) Greuter. J. Tradit. Complement. Med..

[3] Mrid R.B., Bouchmaa N., Kabach I., Zouaoui Z., Chtibi H., El Maadoudi M., Kounnoun A., Cacciola F., El Majdoub Y.O., Mondello L. (2022). *Dittrichia viscosa* L. Leaves: A Valuable Source of Bioactive Compounds with Multiple Pharmacological Effects. Molecules.

[4] Sevgi E., Dag A., Kızılarslan-Hançer Ç., Atasoy S., Kurt B.Z., Aksakal Ö. (2021). Evaluation of Cytotoxic and Antioxidant Potential of *Dittrichia viscosa* (L.) Greuter Used in Traditional Medicine. J. Ethnopharmacol..

[5] Anglana C., Rojas M., Girelli C.R., Barozzi F., Quiroz-Troncoso J., Alegría-Aravena N., Montefusco A., Durante M., Fanizzi F.P., Ramírez-Castillejo C. (2023). Methanolic Extracts of *D. viscosa* Specifically Affect the Cytoskeleton and Exert an Antiproliferative Effect on Human Colorectal Cancer Cell Lines, According to Their Proliferation Rate. Int. J. Mol. Sci..

[6] Rhimi W., Hlel R., Ben Salem I., Boulila A., Rejeb A., Saidi M. (2019). *Dittrichia viscosa* L. Ethanolic Extract Based Ointment with Antiradical, Antioxidant, and Healing Wound Activities. BioMed Res. Int..

[7] Grauso L., Cesarano G., Zotti M., Ranesi M., Sun W., Bonanomi G., Lanzotti V. (2020). Exploring Dittrichia viscosa (L.) Greuter Phytochemical Diversity to Explain Its Antimicrobial, Nematicidal and Insecticidal Activity.

[8] Bentarhlia N., Kartah B.E., Fadil M., El Harkaoui S., Matthäus B., Abboussi O., Abdelmoumen H., Bouhnik O., El Monfalouti H. (2024). Exploring the Wound-Healing and Antimicrobial Potential of *Dittrichia viscosa* L. Lipidic Extract: Chemical Composition and in Vivo Evaluation. Fitoterapia.

[9] Rhimi W., Ben Salem I., Iatta R., Chaabane H., Saidi M., Boulila A., Cafarchia C. (2018). *Dittrichia viscosa* L. Leaves Lipid Extract: An Unexploited Source of Essential Fatty Acids and Tocopherols with Antifungal and Anti-Inflammatory Properties. Ind. Crops Prod..

[10] Segura-Navarro M.J., Quílez del Moral J.F., Andrés M.F., Valcárcel F., González-Coloma A., Molina Inzunza D.O., Barrero A.F. (2025). Major Components of *Dittrichia viscosa* (Asteraceae) as a Source of New Pesticides. Molecules.

[11] Morelli M., De Rosa A., Silvestre G.M., Cennamo P., Pollio A., Cimmino A., Masi M., Carpentieri A. (2026). Screening of *Dittrichia viscosa* (L.) Greuter Metabolites as Potential Natural Biocides for Cultural Heritage Applications. npj Herit. Sci..

[12] Lee C.M., Lee J., Nam M.J., Choi Y.S., Park S.H. (2019). Tomentosin Displays Anti-Carcinogenic Effect in Human Osteosarcoma MG-63 Cells via the Induction of Intracellular Reactive Oxygen Species. Int. J. Mol. Sci..

[13] Rozenblat S., Grossman S., Bergman M., Gottlieb H., Cohen Y., Dovrat S. (2008). Induction of G2/M Arrest and Apoptosis by Sesquiterpene Lactones in Human Melanoma Cell Lines. Biochem. Pharmacol..

[14] Matos M.S., Anastácio J.D., Dos Santos C.N. (2021). Sesquiterpene Lactones: Promising Natural Compounds to Fight Inflammation. Pharmaceutics.

[15] El Omari N., El Menyiy N., Zengin G., Goh B.H., Gallo M., Montesano D., Naviglio D., Bouyahya A. (2021). Anticancer and Anti-Inflammatory Effects of Tomentosin: Cellular and Molecular Mechanisms. Separations.

[16] Migheli R., Virdis P., Galleri G., Arru C., Lostia G., Coradduzza D., Muroni M.R., Pintore G., Podda L., Fozza C. (2022). Antineoplastic Properties by Proapoptotic Mechanisms Induction of *Inula viscosa* and Its Sesquiterpene Lactones Tomentosin and Inuviscolide. Biomedicines.

[17] Çetinkaya S., Güçlü E., Çınar Ayan İ., Vural H., Dursun H.G. (2025). A Sesquiterpene Lactone, Tomentosin, as a Novel Anticancer Agent: Orchestrating Apoptosis, Autophagy, and ER Stress in Colorectal Cancer. Naunyn. Schmiedebergs. Arch. Pharmacol..

[18] Ren W., Bian Y., Zhang Z., Shang H., Zhang P., Chen Y., Yang Z., Luo T., Tang Y. (2012). Enantioselective and Collective Syntheses of Xanthanolides Involving a Controllable Dyotropic Rearrangement of cis-*β*-Lactones. Angew. Chem. Int. Ed..

[19] Kebbi S., Ciavatta M.L., Mahmoud A.M., Carbone M., Ligresti A., Seghiri R., Gavagnin M. (2021). Sesquiterpene Lactones with the 12,8-Guaianolide Skeleton from Algerian *Centaurea omphalotricha*. Biomolecules.

[20] Hyldgaard M.G., Purup S., Bond A.D., Fretté X.C., Qu H., Jensen K.T., Christensen L.P. (2015). Guaianolides and a Seco-Eudesmane from the Resinous Exudates of Cushion Bush (*Leucophyta brownii*) and Evaluation of Their Cytostatic and Anti-Inflammatory Activity. J. Nat. Prod..

[21] Vu Q.V., Sayama S., Ando M., Kataoka T. (2024). Sesquiterpene lactones containing an α-methylene-γ-lactone moiety selectively down-regulate the expression of tumor necrosis factor receptor 1 by promoting its ectodomain shedding in human lung adenocarcinoma A549 cells. Molecules.

[22] Boyd M.R., Padl K.D. (1995). Some Practical Considerations and Applications of the National Cancer Institute In Vitro Anticancer Drug Discovery Screen. Drug Dev. Res..

[23] Geran R.S., Greenberg N.H., Macdonald M.M., Schumacher A.M., Abbott B.J. (1972). Protocols for Screening Chemical Agents and Natural Products against Animal Tumors and Other Biological Systems. Cancer Chemotherapy Reports.

[24] Li Y., Meng Q., Yang M., Liu D., Hou X., Tang L., Wang X., Lyu Y., Chen X., Liu K. (2019). Current Trends in Drug Metabolism and Pharmacokinetics. Acta Pharm. Sin. B.

[25] Guan L., Yang H., Cai Y., Sun L., Di P., Li W., Liu G., Tang Y. (2019). ADMET-Score–a Comprehensive Scoring Function for Evaluation of Chemical Drug-Likeness. Medchemcomm.

[26] Safari-Alighiarloo N., Taghizadeh M., Rezaei-Tavirani M., Goliaei B., Peyvandi A.A. (2014). Protein-Protein Interaction Networks (PPI) and Complex Diseases. Gastroenterol. Hepatol. Bed Bench.

[27] Wang X., Crowe P.J., Goldstein D., Yang J.L. (2012). STAT3 Inhibition, a Novel Approach to Enhancing Targeted Therapy in Human Cancers (Review). Int. J. Oncol..

[28] Kumar A., Bora U. (2012). Molecular Docking Studies on Inhibition of Stat3 Dimerization by Curcumin Natural Derivatives and Its Conjugates with Amino Acids. Bioinformation.

[29] Bai L., Zhou H., Xu R., Zhao Y., Chinnaswamy K., McEachern D., Chen J., Yang C.Y., Liu Z., Wang M. (2019). A Potent and Selective Small-Molecule Degrader of STAT3 Achieves Complete Tumor Regression In Vivo. Cancer Cell.

[30] Shiau A.K., Barstad D., Loria P.M., Cheng L., Kushner P.J., Agard D.A., Greene G.L. (1998). The Structural Basis of Estrogen Receptor/Coactivator Recognition and the Antagonism of This Interaction by Tamoxifen. Cell.

[31] Orlando B.J., Malkowski M.G. (2016). Crystal Structure of Rofecoxib Bound to Human Cyclooxygenase-2. Acta Crystallogr. Sect. F Struct. Biol. Commun..

[32] Hawash M., Jaradat N., Abualhasan M., Şüküroğlu M.K., Qaoud M.T., Kahraman D.C., Daraghmeh H., Maslamani L., Sawafta M., Ratrout A. (2023). Design, Synthesis, Molecular Docking Studies and Biological Evaluation of Thiazole Carboxamide Derivatives as COX Inhibitors. BMC Chem..

[33] Leão R.P., Cruz J.V., da Costa G.V., Cruz J.N., Ferreira E.F.B., Silva R.C., de Lima L.R., Borges R.S., Dos Santos G.B., Santos C.B.R. (2020). Identification of New Rofecoxib-Based Cyclooxygenase-2 Inhibitors: A Bioinformatics Approach. Pharmaceuticals.

[34] Md Idris M.H., Mohd Amin S.N., Mohd Amin S.N., Nyokat N., Khong H.Y., Selvaraj M., Zakaria Z.A., Shaameri Z., Hamzah A.S., Teh L.K. (2022). Flavonoids as Dual Inhibitors of Cyclooxygenase-2 (COX-2) and 5-Lipoxygenase (5-LOX): Molecular Docking and in Vitro Studies. Beni. Suef. Univ. J. Basic Appl. Sci..

[35] Chadwick M., Trewin H., Gawthrop F., Wagstaff C. (2013). Sesquiterpenoids Lactones: Benefits to Plants and People. Int. J. Mol. Sci..

[36] Jackson P.A., Widen J.C., Harki D.A., Brummond K.M. (2017). Covalent Modifiers: A Chemical Perspective on the Reactivity of α,β-Unsaturated Carbonyls with Thiols via Hetero-Michael Addition Reactions. J. Med. Chem..

[37] Karplus M., Kuriyan J. (2005). Molecular Dynamics and Protein Function. Proc. Natl. Acad. Sci. USA.

[38] Hu A., Chen H., Pang W., Pu X., Qi Z., Chen H. (2024). Identification of Potential Modulators for Human GPD1 by Docking-Based Virtual Screening, Molecular Dynamics Simulations, Binding Free Energy Calculations, and DeLA-Drug Analysis. Sci. Rep..

[39] Rampogu S., Lee G., Park J.S., Lee K.W., Kim M.O. (2022). Molecular Docking and Molecular Dynamics Simulations Discover Curcumin Analogue as a Plausible Dual Inhibitor for SARS-CoV-2. Int. J. Mol. Sci..

[40] Suleman M., Moltrasio C., Tricarico P.M., Marzano A.V., Crovella S. (2024). Natural Compounds Targeting Thymic Stromal Lymphopoietin (TSLP): A Promising Therapeutic Strategy for Atopic Dermatitis. Biomolecules.

[41] Fatriansyah J.F., Boanerges A.G., Kurnianto S.R., Pradana A.F., Fadilah, Surip S.N. (2022). Molecular Dynamics Simulation of Ligands from *Anredera cordifolia* (Binahong) to the Main Protease (Mpro) of SARS-CoV-2. J. Trop. Med..

[42] Ghufran M., Khan H.A., Ullah M., Ghufran S., Ayaz M., Siddiq M., Shams S., Bungau S. (2022). In Silico Strategies for Designing of Peptide Inhibitors of and Proliferation. Cancers.

[43] Karousa M.M., Kalath H., Karam L., Suleman M., Ayoub M.M., Fathima A., Rocha M.A.M., Mechmechani S., Pinto D.C.G.A., Yassine H.M. (2026). *Chrysopogon zizanioides* (Vetiver) Essential Oil from Qatar Targets AKT1 and STAT3 in Colorectal and Lung Cancer: GC-MS Profiling, In Vitro Antiproliferative Activity, and In Silico Analyses. Plants.

[44] Seglab F., Abou Assali M., AlYafei T., Hassan H., Pinto D.C.G.A., Baydoun S., Al Thani A.A., Shaito A.A. (2024). Chemical Composition, Antioxidant Capacity, and Anticancerous Effects against Human Lung Cancer Cells of a Terpenoid-Rich Fraction of *Inula viscosa*. Biology.

[45] Daina A., Michielin O., Zoete V. (2017). SwissADME: A Free Web Tool to Evaluate Pharmacokinetics, Drug-Likeness and Medicinal Chemistry Friendliness of Small Molecules. Sci. Rep..

[46] Pires D.E.V., Blundell T.L., Ascher D.B. (2015). PkCSM: Predicting Small-Molecule Pharmacokinetic and Toxicity Properties Using Graph-Based Signatures. J. Med. Chem..

[47] Daina A., Michielin O., Zoete V. (2019). SwissTargetPrediction: Updated Data and New Features for Efficient Prediction of Protein Targets of Small Molecules. Nucleic Acids Res..

[48] Pogodin P.V., Lagunin A.A., Filimonov D.A., Poroikov V.V. (2015). PASS Targets: Ligand-Based Multi-Target Computational System Based on a Public Data and Naïve Bayes Approach. SAR QSAR Environ. Res..

[49] Szklarczyk D., Gable A.L., Nastou K.C., Lyon D., Kirsch R., Pyysalo S., Doncheva N.T., Legeay M., Fang T., Bork P. (2021). The STRING Database in 2021: Customizable Protein–Protein Networks, and Functional Characterization of User-Uploaded Gene/Measurement Sets. Nucleic Acids Res..

[50] Doncheva N.T., Morris J.H., Holze H., Kirsch R., Nastou K.C., Cuesta-Astroz Y., Rattei T., Szklarczyk D., von Mering C., Jensen L.J. (2023). Cytoscape StringApp 2.0: Analysis and Visualization of Heterogeneous Biological Networks. J. Proteome Res..

[51] Lotia S., Montojo J., Dong Y., Bader G.D., Pico A.R. (2013). Cytoscape App Store. Bioinformatics.

[52] Chin C.H., Chen S.H., Wu H.H., Ho C.W., Ko M.T., Lin C.Y. (2014). CytoHubba: Identifying Hub Objects and Sub-Networks from Complex Interactome. BMC Syst. Biol..

[53] Kanehisa M., Furumichi M., Sato Y., Ishiguro-Watanabe M., Tanabe M. (2021). KEGG: Integrating Viruses and Cellular Organisms. Nucleic Acids Res..

[54] Ge S.X., Jung D., Jung D., Yao R. (2020). ShinyGO: A Graphical Gene-Set Enrichment Tool for Animals and Plants. Bioinformatics.

[55] Luo W., Brouwer C. (2013). Pathview: An R/Bioconductor Package for Pathway-Based Data Integration and Visualization. Bioinformatics.

[56] Eberhardt J., Santos-Martins D., Tillack A.F., Forli S. (2021). AutoDock Vina 1.2. 0: New Docking Methods, Expanded Force Field, and Python Bindings. J. Chem. Inf. Model..

[57] Trott O., Olson A.J. (2010). AutoDock Vina: Improving the Speed and Accuracy of Docking with a New Scoring Function, Efficient Optimization, and Multithreading. J. Comput. Chem..

[58] Saur I.M.L., Panstruga R., Schulze-Lefert P. (2021). NOD-like Receptor-Mediated Plant Immunity: From Structure to Cell Death. Nat. Rev. Immunol..

[59] Case D.A., Cheatham T.E., Darden T., Gohlke H., Luo R., Merz K.M., Onufriev A., Simmerling C., Wang B., Woods R.J. (2005). The Amber Biomolecular Simulation Programs. J. Comput. Chem..

[60] Salomon-Ferrer R., Case D.A., Walker R.C. (2013). An Overview of the Amber Biomolecular Simulation Package. Wiley Interdiscip. Rev. Comput. Mol. Sci..

[61] Toukmaji A., Sagui C., Board J., Darden T. (2000). Efficient Particle-Mesh Ewald Based Approach to Fixed and Induced Dipolar Interactions. J. Chem. Phys..

[62] Götz A.W., Williamson M.J., Xu D., Poole D., Le Grand S., Walker R.C. (2012). Routine Microsecond Molecular Dynamics Simulations with AMBER on GPUs. 2. Explicit Solvent Particle Mesh Ewald. J. Chem. Theory Comput..

[63] Roe D.R., Cheatham T.E. (2013). PTRAJ and CPPTRAJ: Software for Processing and Analysis of Molecular Dynamics Trajectory Data. J. Chem. Theory Comput..

[64] Maiorov V.N., Crippen G.M. (1994). Significance of Root-Mean-Square Deviation in Comparing Three-Dimensional Structures of Globular Proteins. J. Mol. Biol..

[65] Cooper A. (1976). Thermodynamic Fluctuations in Protein Molecules. Proc. Natl. Acad. Sci. USA.

[66] Genheden S., Ryde U. (2015). The MM/PBSA and MM/GBSA Methods to Estimate Ligand-Binding Affinities. Expert Opin. Drug Discov..

[67] Sun H., Duan L., Chen F., Liu H., Wang Z., Pan P., Zhu F., Zhang J.Z.H., Hou T. (2018). Assessing the Performance of MM/PBSA and MM/GBSA Methods. 7. Entropy Effects on the Performance of End-Point Binding Free Energy Calculation Approaches. Phys. Chem. Chem. Phys..

