# Supplementary material for: In Vitro and In Silico Insights into the Antiproliferative Activity of Sesquiterpenes from Dittrichia viscosa (L.) Greuter
Source: Int J Mol Sci. 2026 Aug 6;27(15):7064. doi: 10.3390/ijms27157064 (PMC13467573; doi:10.3390/ijms27157064)
Supplement: Supplementary file 1 [file ijms-27-07064-s001.zip › supporting material_REV2.pdf]

Supplementary Information

In Vitro and In Silico Insights into the Antiproliferative Activity of Sesquiterpenes from *Dittrichia viscosa* (L.) Greuter

Maria Michela Salvatore[1], Marco Masi[2], Muhammad Suleman[3], Haritha Kalath[3], Mai M. Karousa[3,4], Maha M. Ayoub[3,4], Abdullah A. Shaito[3,4,5*], Anna Andolfi[2,*]

[1] Department of Veterinary Medicine and Animal Production, University of Naples Federico II, Naples, Italy; mariamichela.salvatore@unina.it (M.M.S.)

[2] Department of Chemical Science, University of Naples Federico II, Naples, Italy; marco.masi@unina.it (M.M.)

[3] Biomedical Research Center (BRC), QU Health, Qatar University, Doha P.O. Box 2713, Qatar; m.suleman@qu.edu.qa (M.S.); harithakalath@gmail.com (H.K.)

[4] Department of Biomedical Sciences, College of Health Sciences, QU Health, Qatar University, Doha P.O. Box 2713, Qatar; mk2200561@student.qu.edu.qa (M.M.K.); ma2400339@student.qu.edu.qa (M.M.A.)

[5] College of Medicine, Qatar University, QU Health, Qatar University, Doha P.O. Box 2713, Qatar

* Correspondence: abdshaito@qu.edu.qa (A.A.S.); andolfi@unina.it (A.A.)

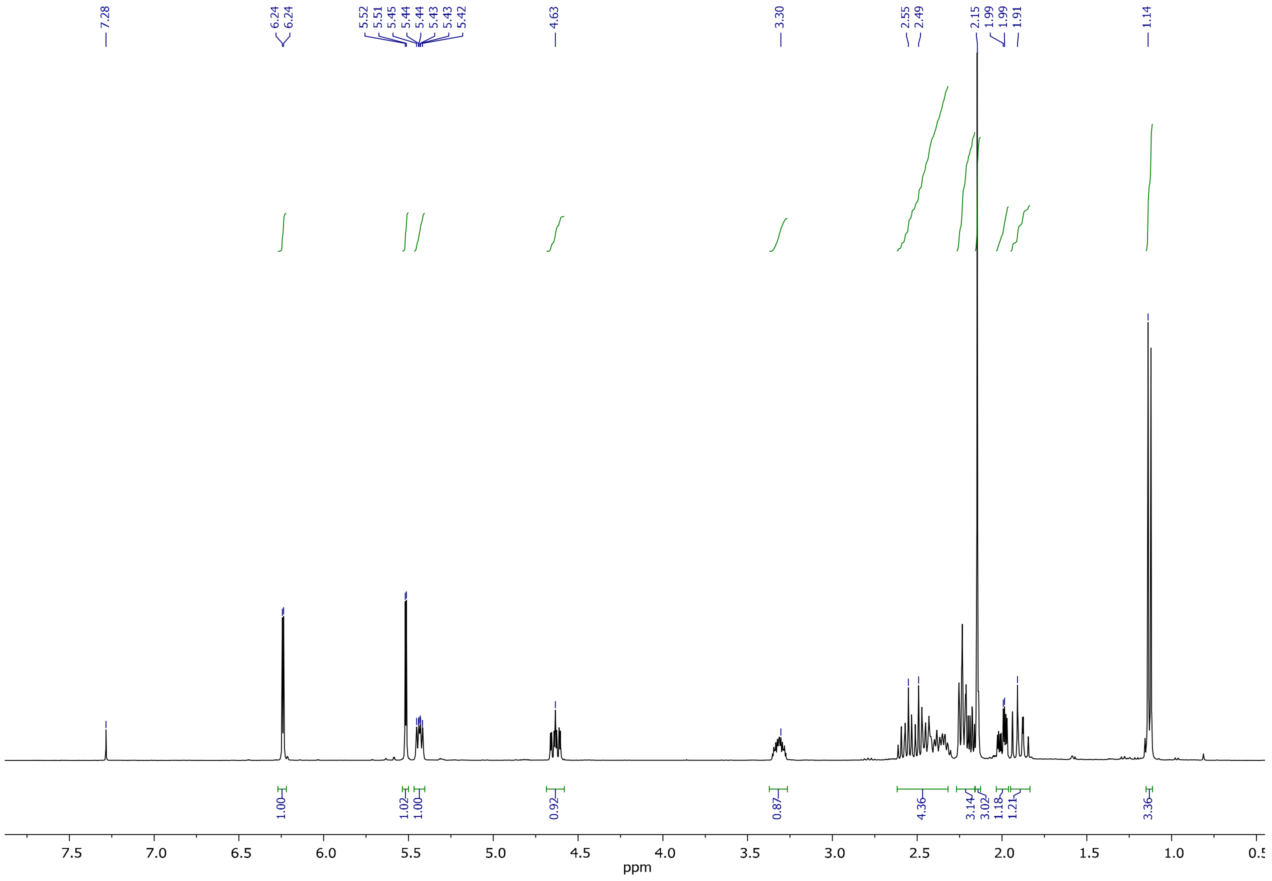

Figure S1. ^{1}H NMR spectrum of tomentosin recorded in $CDCl_3$ at 400 MHz.

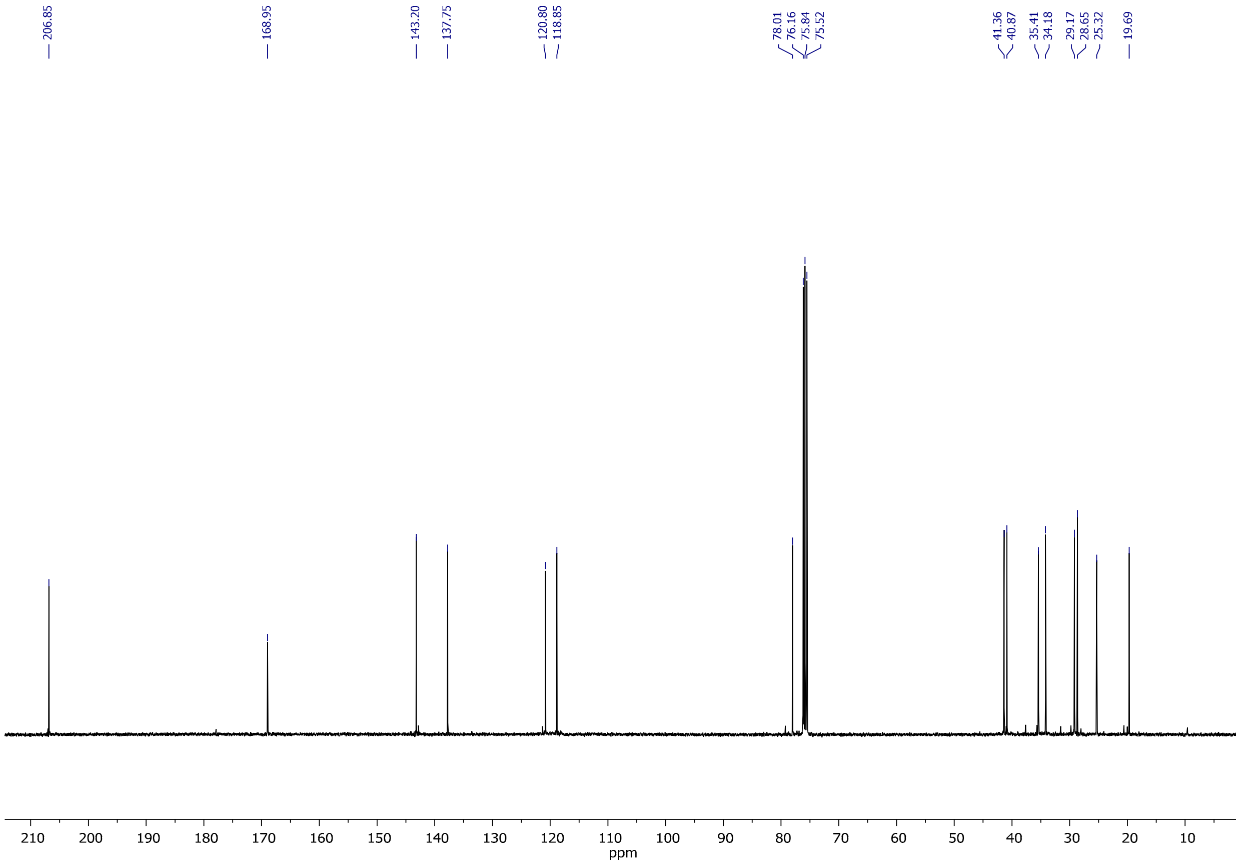

Figure S2. ^{13}C NMR spectrum of tomentosin recorded in $CDCl_3$ at 100 MHz.

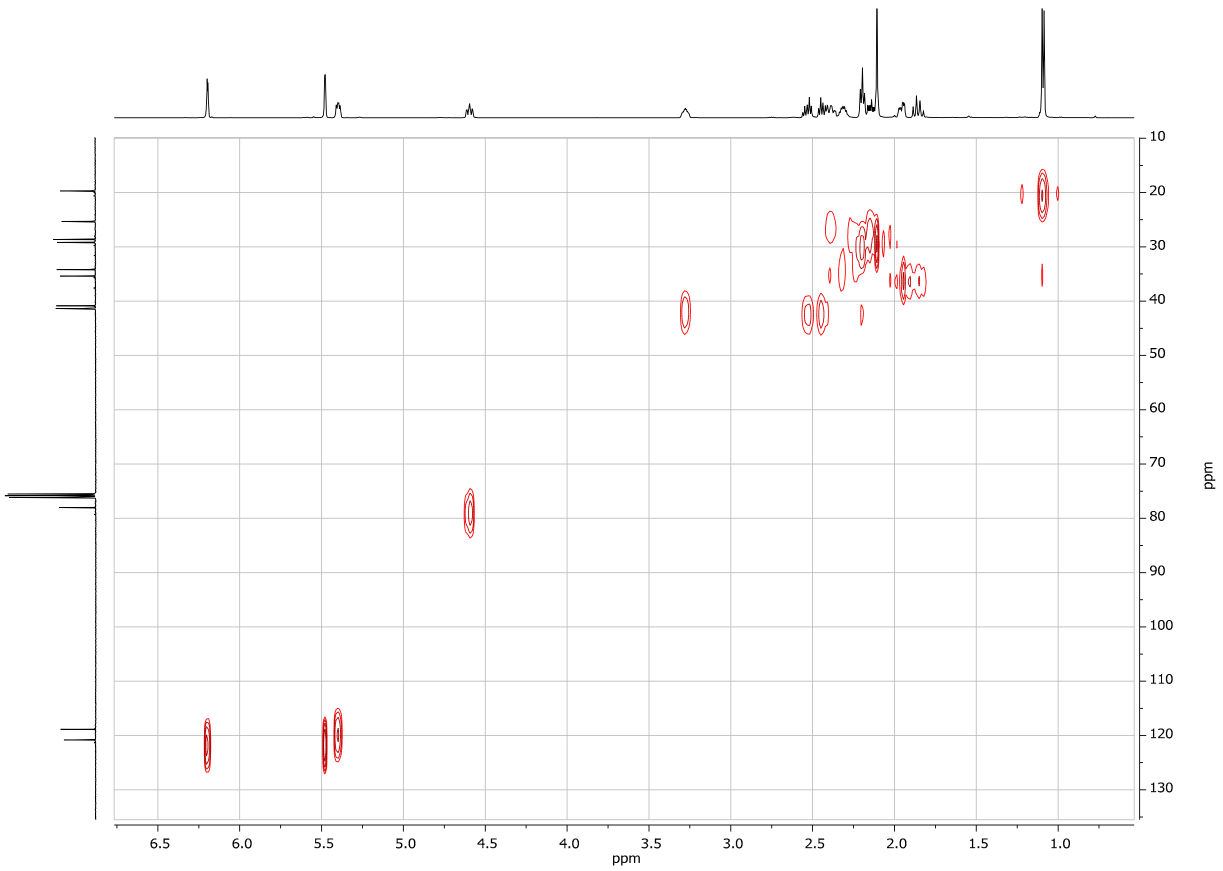

Figure S3. HSQC spectrum of tomentosin recorded in $CDCl_3$.

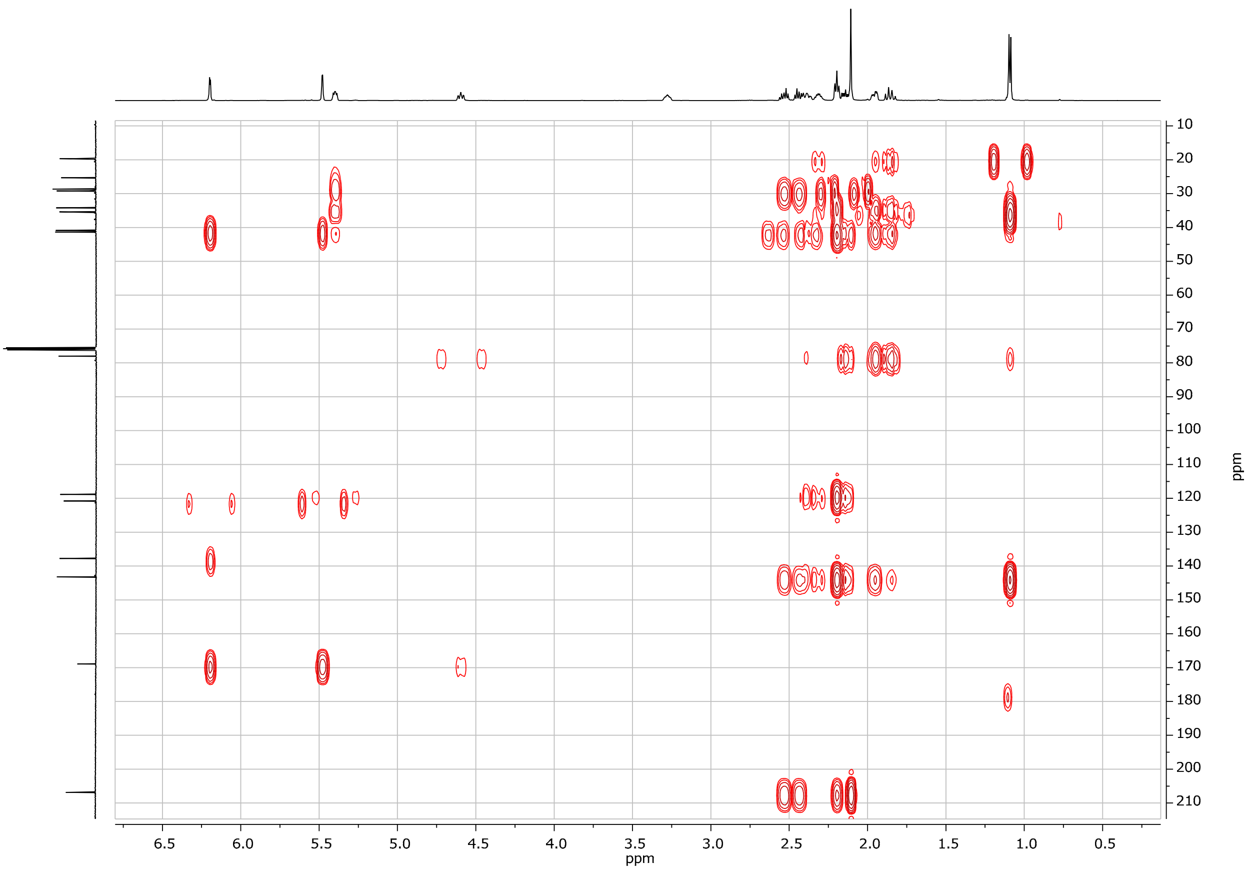

Figure S4. HMBC spectrum of tomentosin recorded in $CDCl_3$.

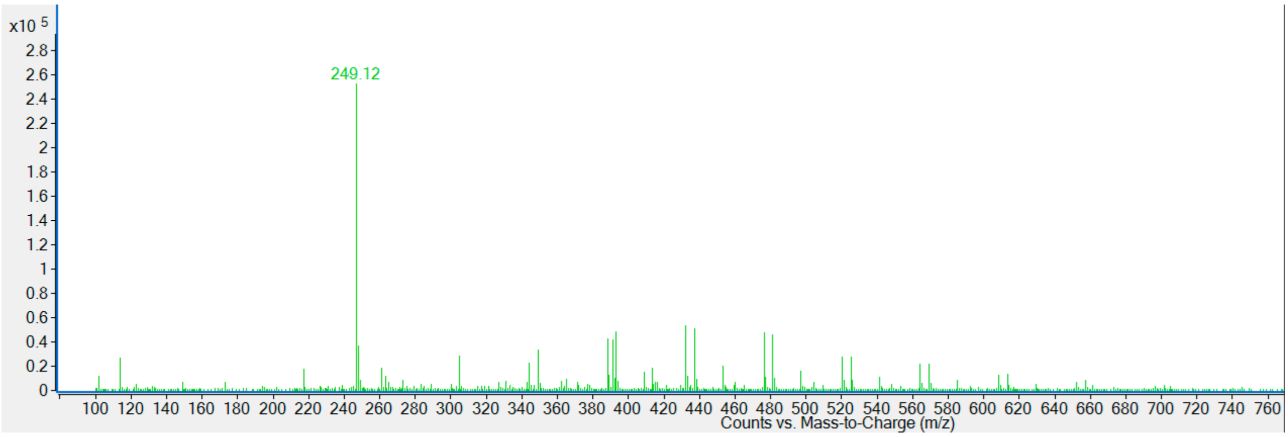

Figure S5. ESI MS spectrum of tomentosin, recorded in positive mode.

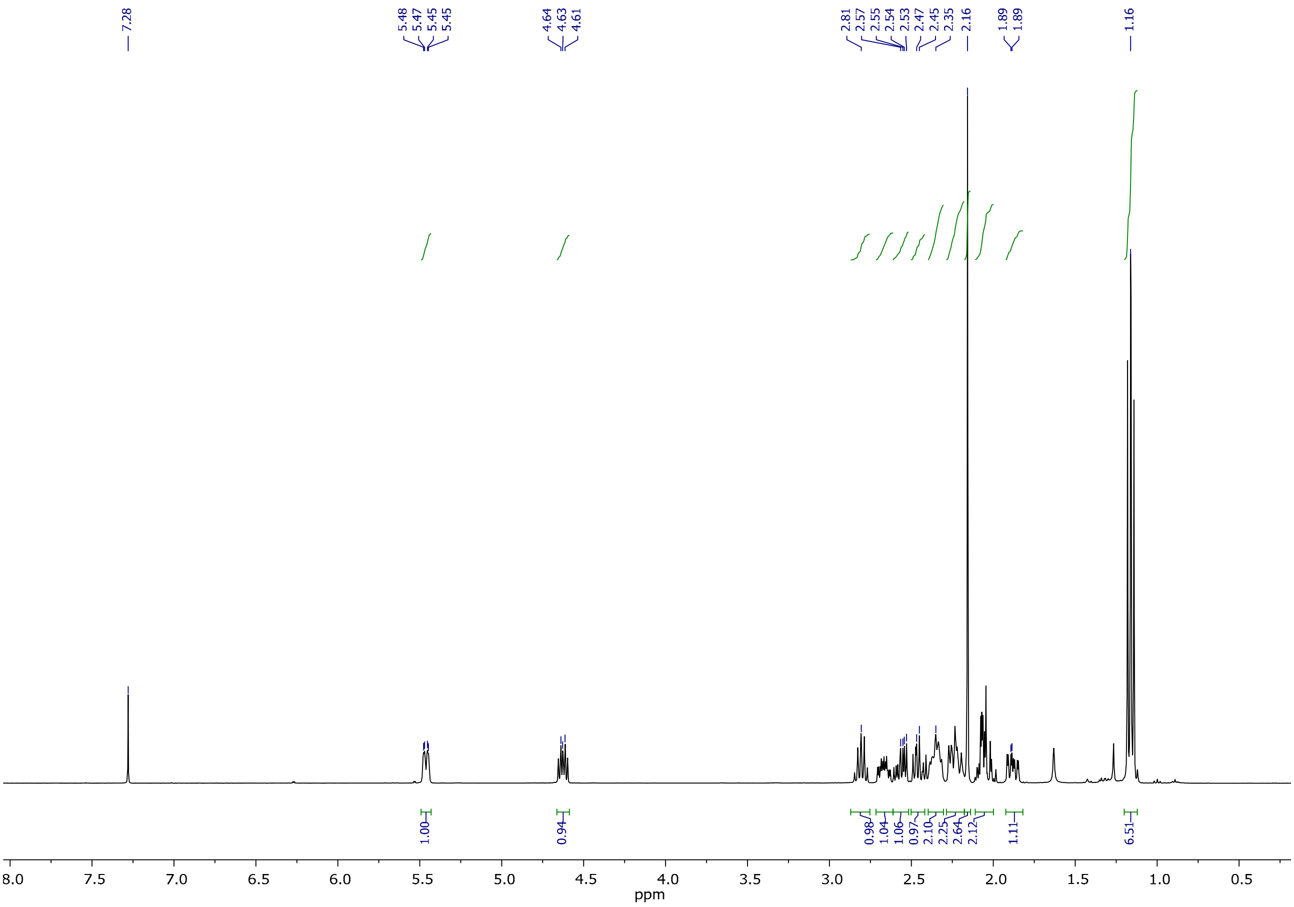

Figure S6. ^{1}H NMR spectrum of 11α,13-dihydrotomentosin recorded in $CDCl_3$ at 400 MHz.

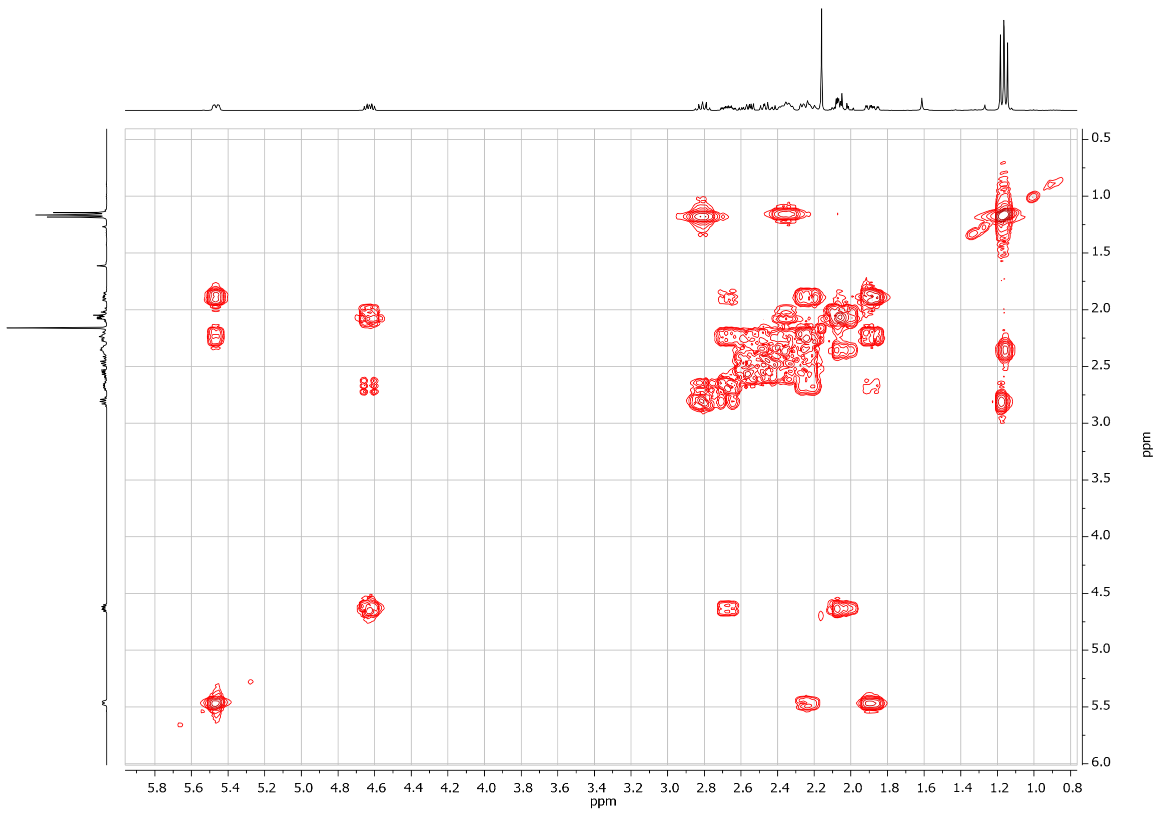

Figure S7. COSY spectrum of 11α,13-dihydrotomentosin recorded in $CDCl_3$.

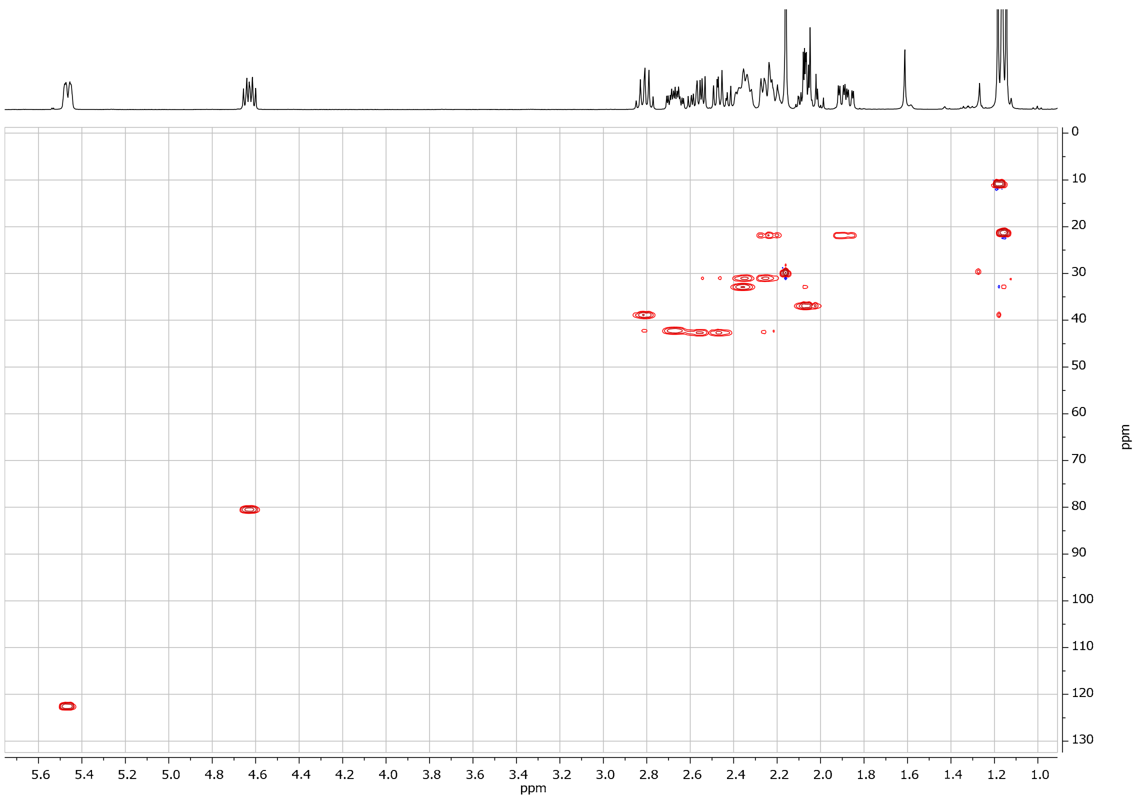

Figure S8. HSQC spectrum of 11α,13-dihydrotomentosin recorded in $CDCl_3$.

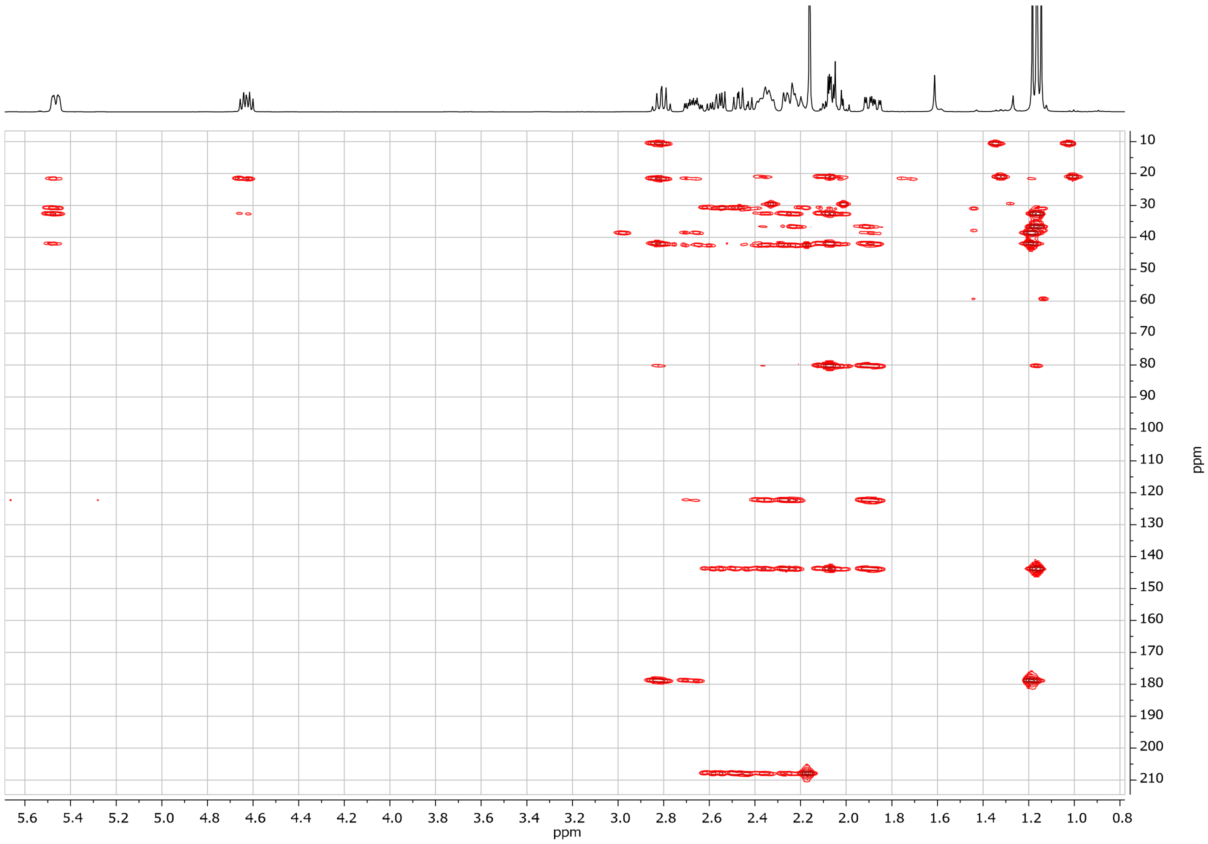

Figure S9. HMBC spectrum of 11α,13-dihydrotomentosin recorded in $CDCl_3$.

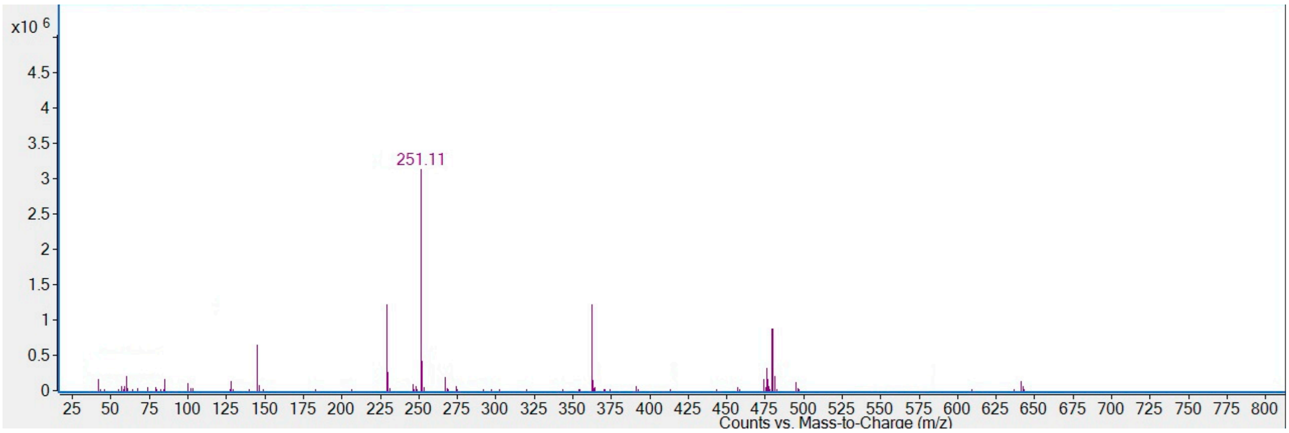

Figure S10. ESI MS spectrum of 11α,13-dihydrotomentosin, recorded in positive mode.

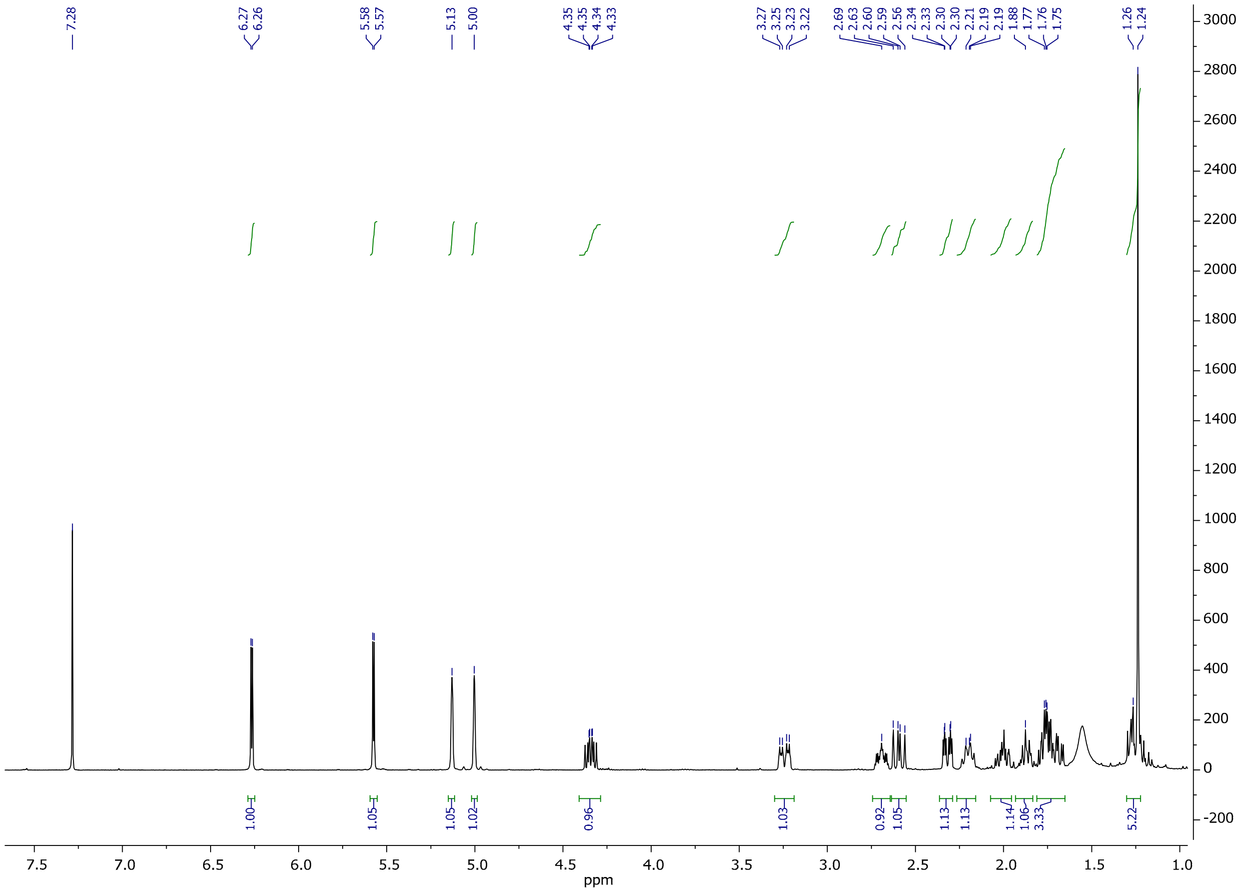

Figure S11. ^{1}H NMR spectrum of inuviscolide recorded in $CDCl_3$ at 400 MHz.

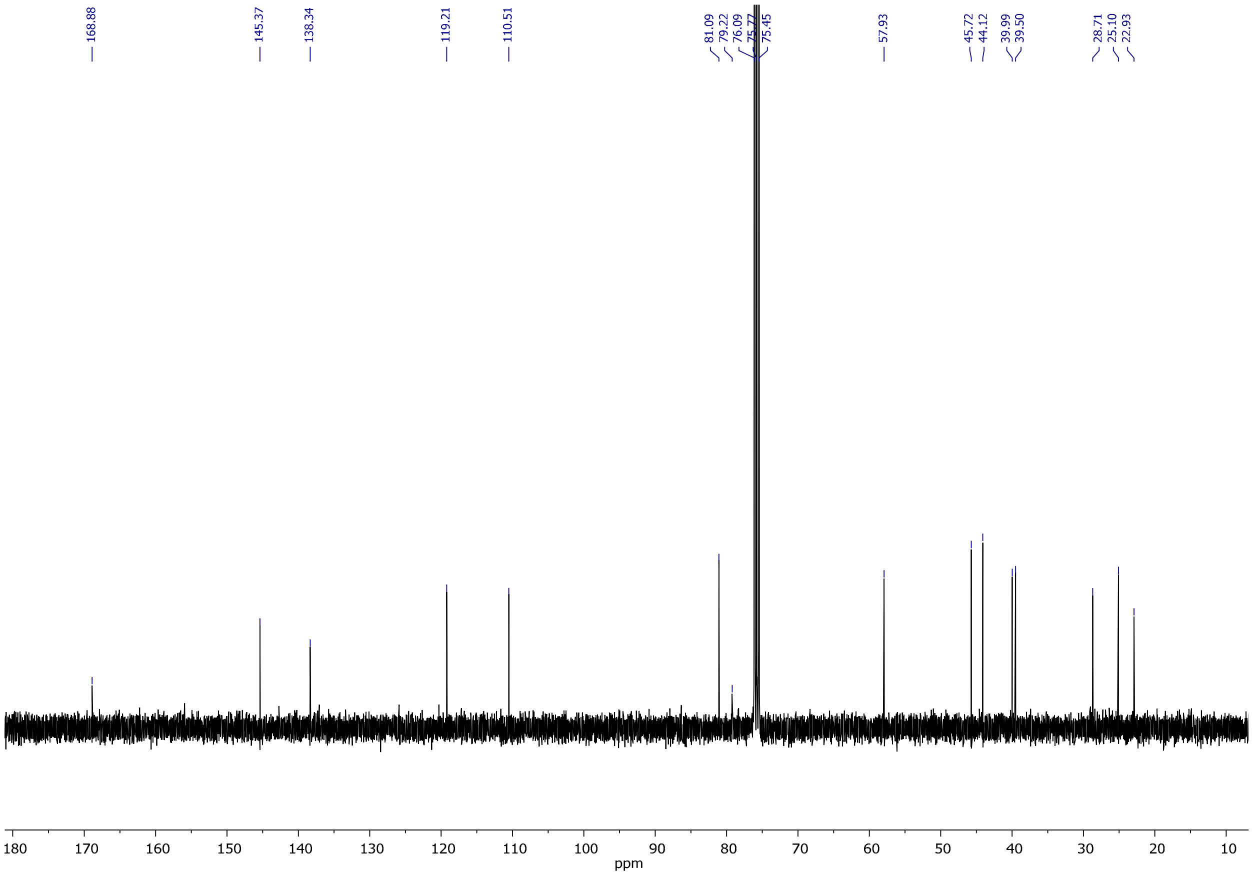

Figure S12. ^{13}C NMR spectrum of inuviscolide recorded in $CDCl_3$ at 100 MHz.

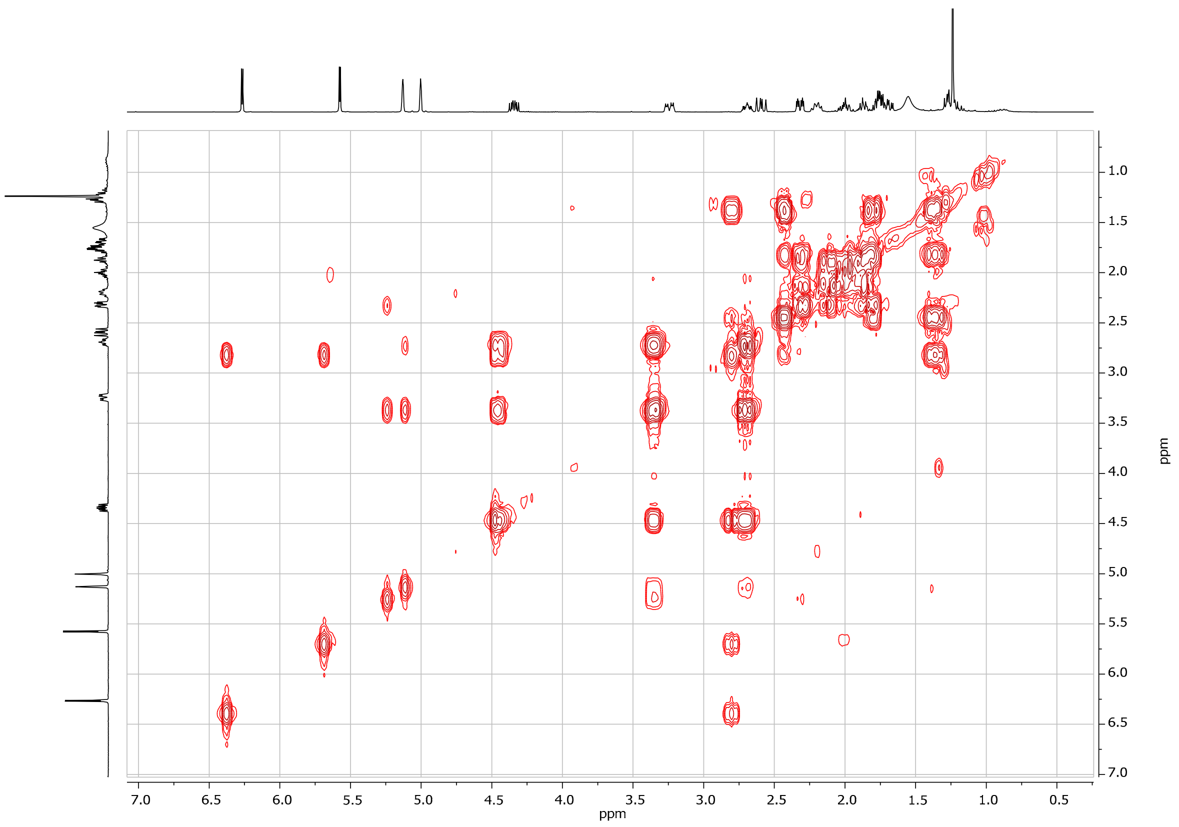

Figure S13. COSY spectrum of inuviscolide recorded in $CDCl_3$.

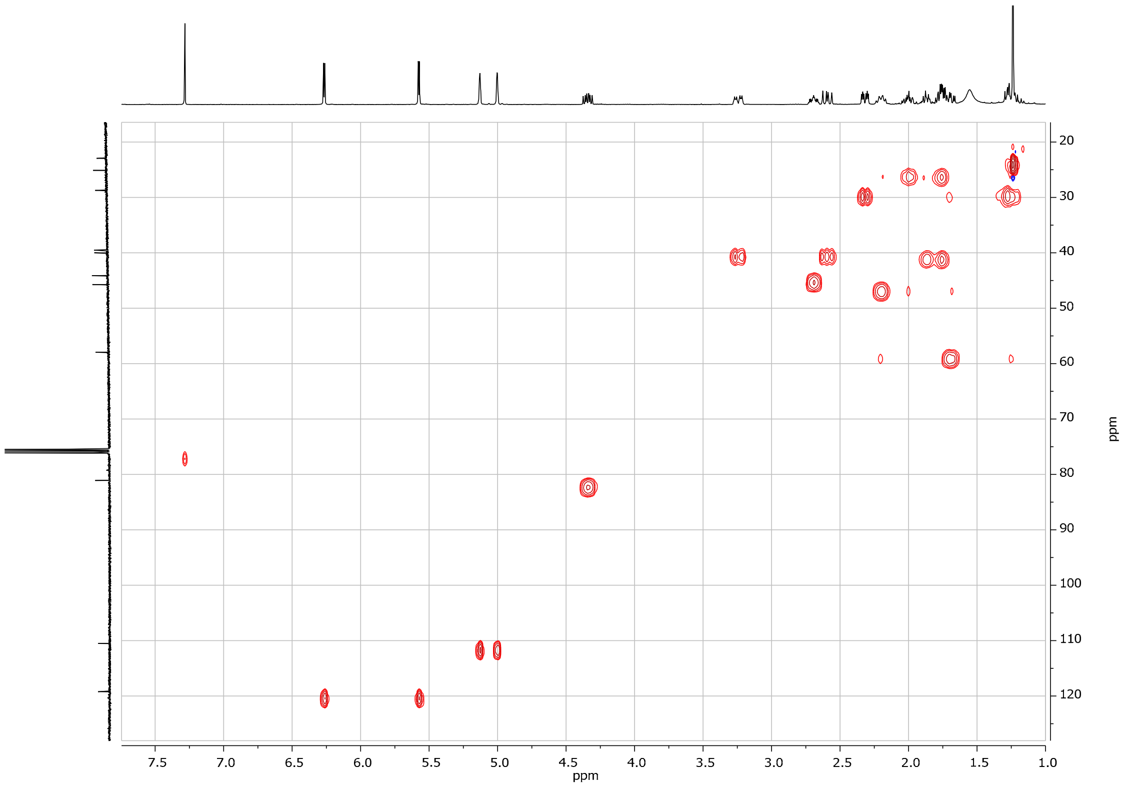

Figure S14. HSQC spectrum of inuviscolide recorded in $CDCl_3$.

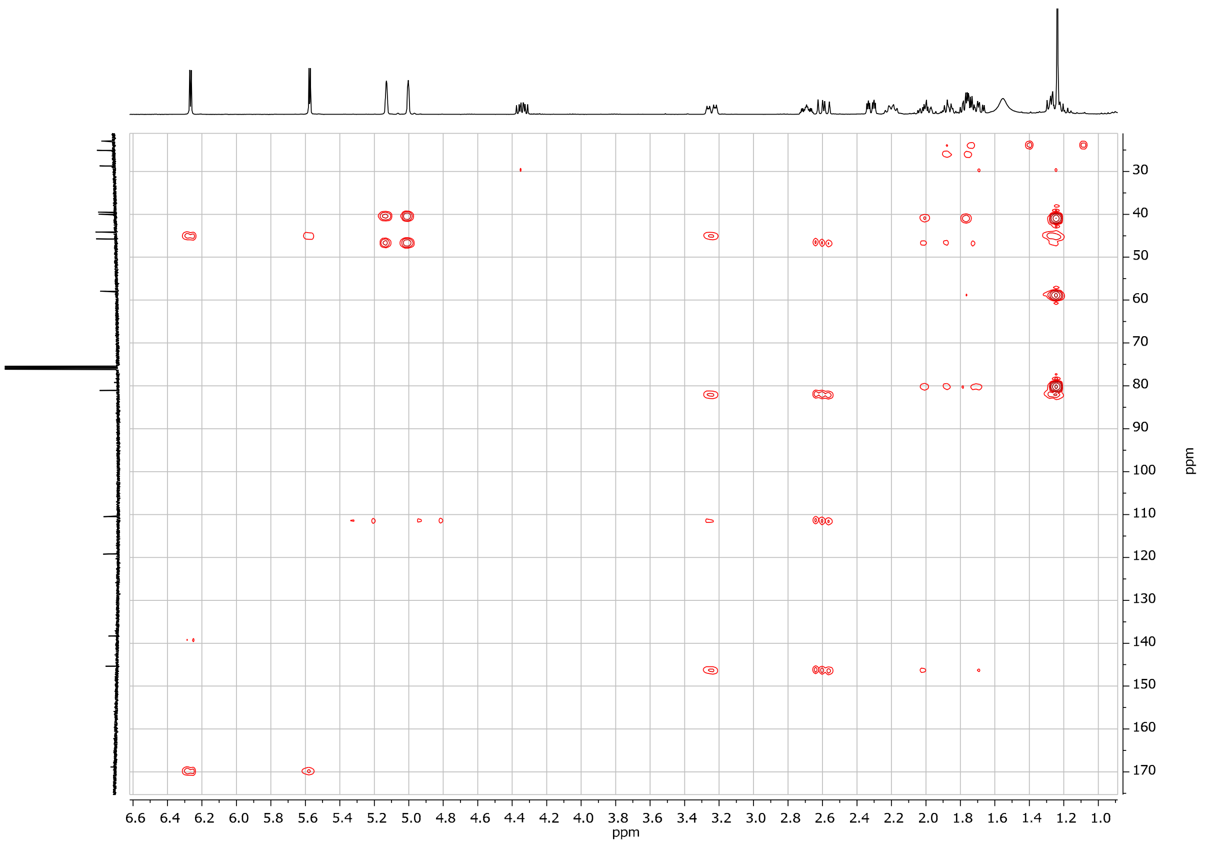

Figure S15. HMBC spectrum of inuviscolide recorded in $CDCl_3$.

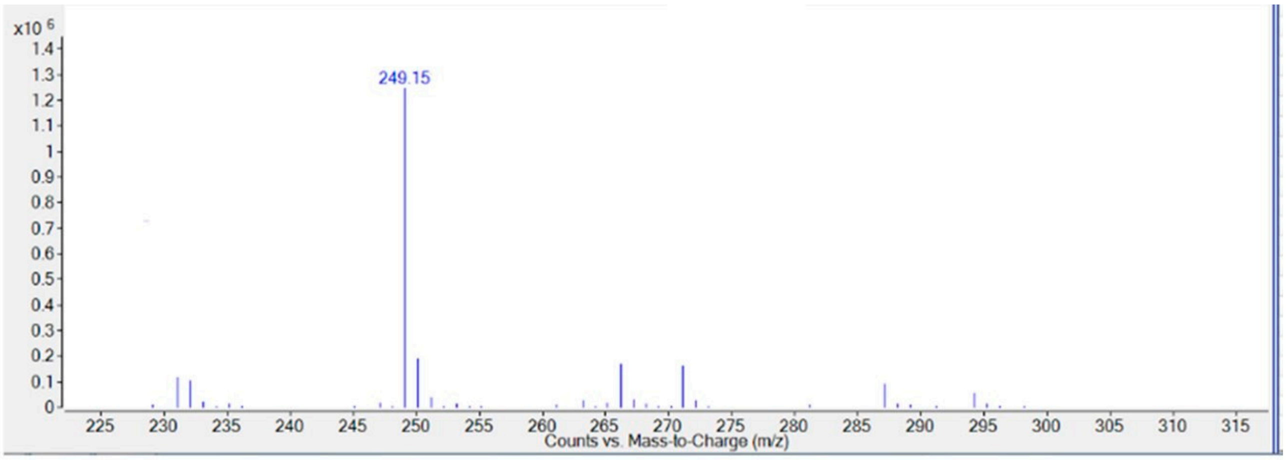

Figure S16. ESI MS spectrum of inuviscolide, recorded in positive mode.

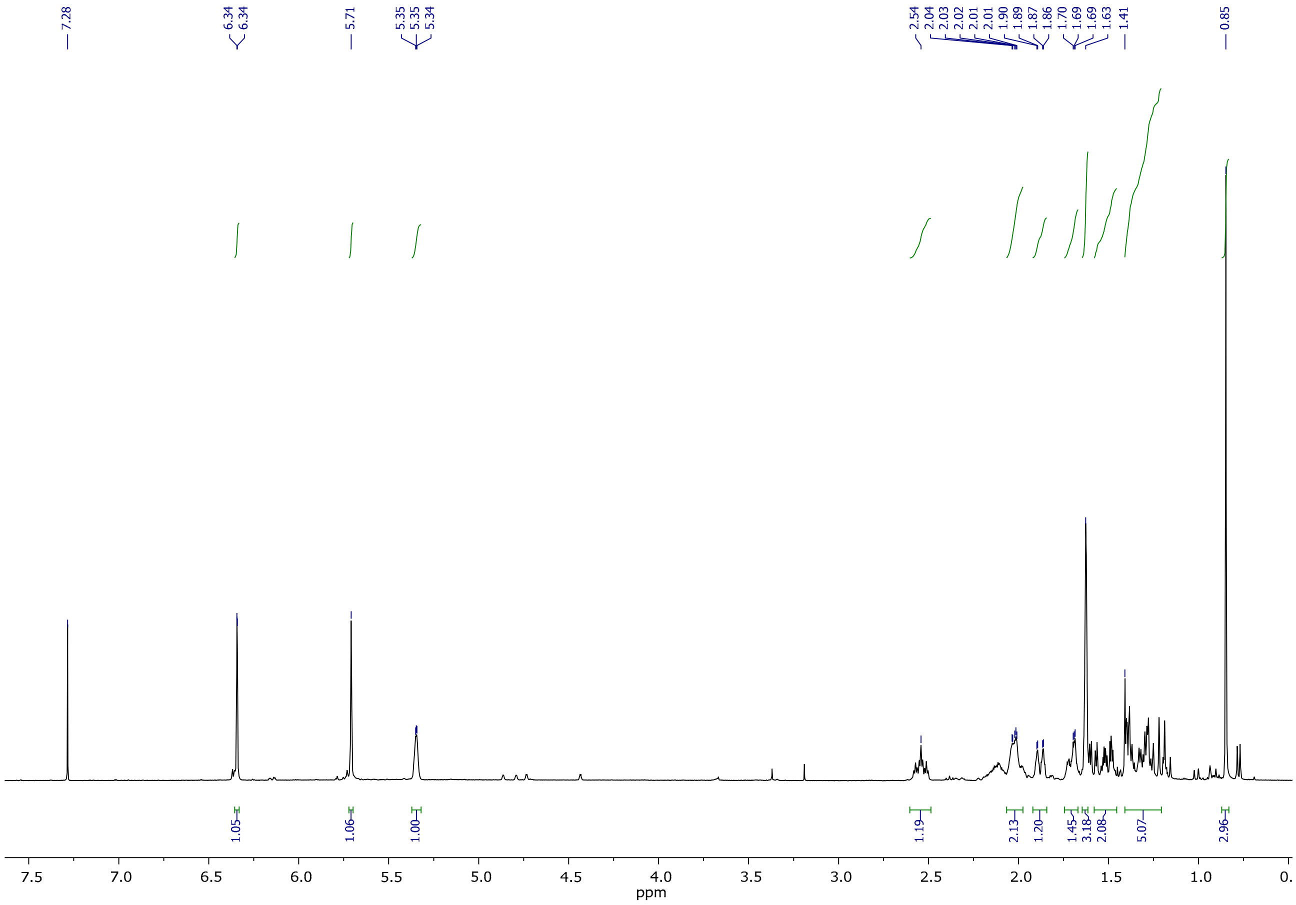

Figure S17. ^{1}H NMR spectrum of α-costic acid recorded in $CDCl_3$ at 400 MHz.

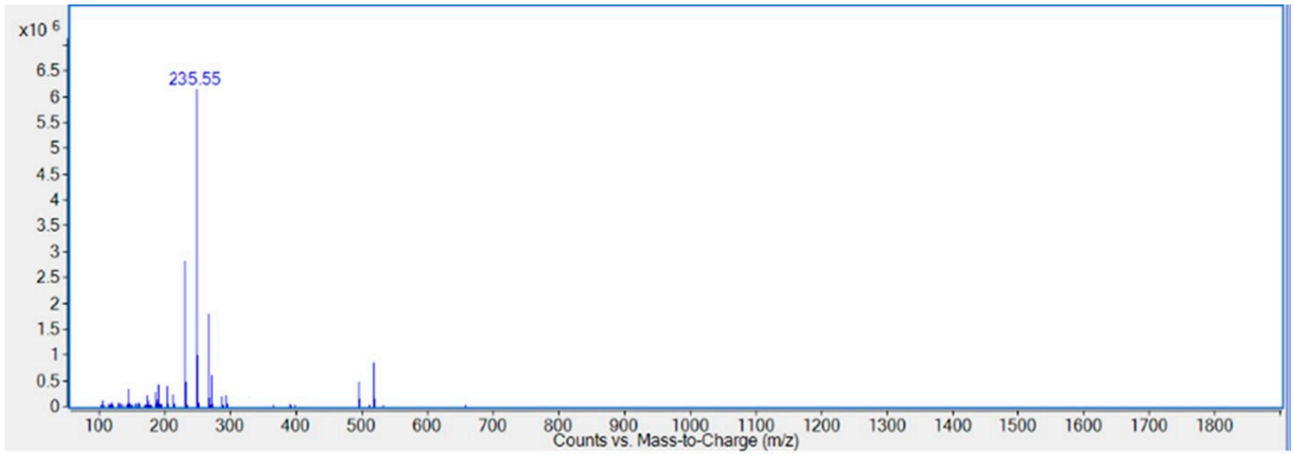

Figure S18. ESI MS spectrum of α-costic acid, recorded in positive mode.

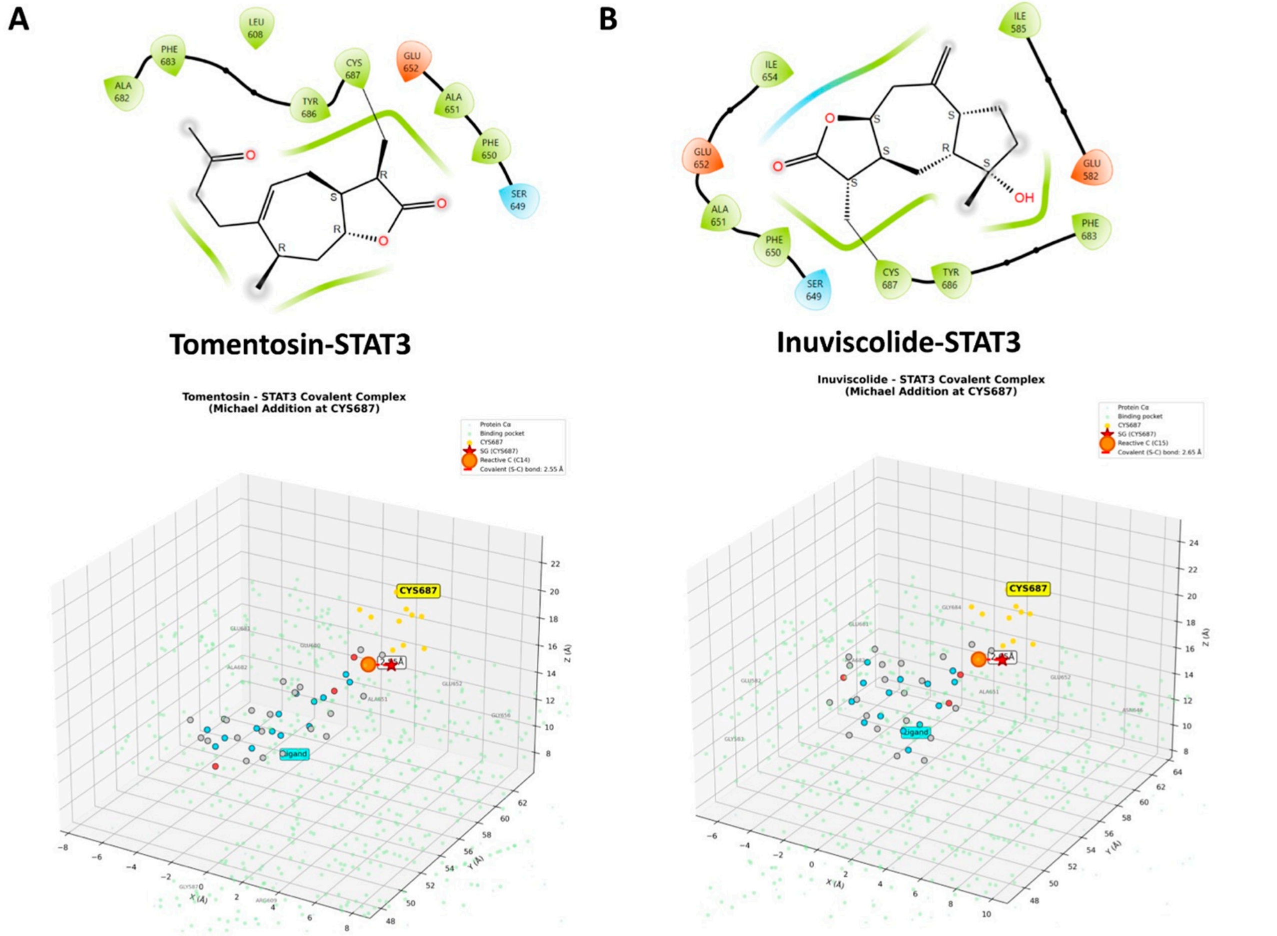

Figure S19. Covalent docking of tomentosin and inuviscolide with CYS687 in the STAT3 SH2 domain. (A) STAT3–tomentosin and (B) STAT3–inuviscolide complexes. Upper panels show two-dimensional interaction maps of the modeled CYS687-linked poses, while lower panels show three-dimensional views of the binding region and surrounding residues.
